# Ketamine for substance use disorders: a systematic review and meta-analysis

**DOI:** 10.3389/fpsyt.2026.1835709

**Published:** 2026-07-17

**Authors:** Shu-Ping Fang, Xin Yang, Da-Cong Zhao, Yang Wen, Yi-Hao Liu, Zhe Li, Mao-Sheng Ran

**Affiliations:** 1 Mental Health Center, West China Hospital, Sichuan University, Chengdu, Sichuan, China; 2 National Center for Mental Disorders, West China Hospital, Sichuan University, Chengdu, Sichuan, China; 3 Department of Social Psychiatry, West China Hospital, Sichuan University, Chengdu, Sichuan, China; 4 Department of Radiology, Dazhou Hospital of Integrated Traditional Chinese and Western Medicine, Dazhou, China

**Keywords:** efficacy, ketamine, meta-analysis, safety, substance use disorder

## Abstract

**Introduction:**

Substance use disorder (SUD) is a major global health issue, contributing significantly to the disease burden. Despite this, effective treatments for SUD remain limited. Ketamine has shown potential as a treatment, yet no meta-analysis has assessed its efficacy for SUD. This study aimed to evaluate the efficacy and safety of ketamine in treating SUD.

**Methods:**

Multiple databases were systematically searched forrandomized controlled trials (RCTs) on ketamine for SUD treatment. The primary outcome was abstinence rates. Adverse events and dropout rates were also assessed to evaluate safety and acceptability. A random-effects model was used to conduct the meta-analysis.

**Results:**

Fifteen RCTs with 798 participants were included; seven RCTs contributed abstinence data to the quantitative synthesis. Ketamine was associated with significantly improved abstinence at <1 month [odds ratio(OR) = 3.27, 95% CI: 1.55–6.92, I^2^ = 0%, *p* < 0.01], but not at 1–6 months (OR = 1.74, 95% CI: 0.91–3.30, I^2^ = 0%, *p* = 0.09). No significant difference in dropout rates was observed between the ketamine and control groups (OR = 0.74, 95% CI: 0.46–1.21, I^2^ = 0%, *p* = 0.23). No significant between group difference was identified in adverse events, although the available evidence was limited and insufficient to establish safety conclusively.

**Discussion:**

Current evidence suggests a short-term efficacy signal for ketamine in SUD, but the evidence remains limited and insufficient to establish its efficacy and safety conclusively.

**Systematic review registration:**

identifier CRD42024607116.

## Introduction

Substance use disorder (SUD) is among the most prevalent mental health conditions (1, 2). The lifetime prevalence of SUD is approximately 10%, while the 12-month prevalence stands at 4% (2). Globally, an estimated 147.5 million people are affected by SUD (2). These disorders are classified based on the substances involved, including commonly used substances such as alcohol, cannabis, opioids, cocaine, stimulants, and sedative–hypnotics (3). Among these, alcohol use disorder (AUD) is the most prevalent (4). In recent years, SUD has become a serious and increasingly widespread global issue (5). However, only a small proportion of individuals with SUD receive treatment (6). The treatment of SUD currently includes approaches such as psychotherapy and pharmacotherapy (7). Although psychosocial interventions have proven effective, relapse remains a frequent occurrence (8). The field of SUD treatment has increasingly turned towards medication-assisted treatment (MAT), primarily due to its demonstrated success in reducing both substance use and harmful behaviors associated with various SUDs (7). There are three U.S. Food and Drug Administration (FDA)-approved medications for treating AUD: disulfiram, acamprosate, and naltrexone (9). For tobacco use disorder (TUD), nicotine replacement therapies (NRTs) are among the most widely used treatments (10). Opioid use disorder (OUD) is commonly treated with opioid agonist and partial agonist therapies, including methadone and buprenorphine, respectively (7). However, no pharmaceutical products have received market authorization for the treatment of amphetamine use disorder (AmUD), cannabis use disorder (CaUD), or cocaine use disorder (CoUD) (9). Furthermore, relapse rates among patients undergoing treatment remain high, with approximately 40% to 60% of individuals experiencing a recurrence of substance use (9).

It is worth mentioning that SUD not only leads to physical health damage but also causes cognitive and behavioral impairments, along with emotional disorders such as anxiety, depression, and suicidal behaviors, which are prevalent in SUD patients (5, 11, 12). Moreover, SUD is more prevalent in individuals with severe mental illnesses than in the general population (13). The presence of comorbid mental health disorders is often associated with poor treatment outcomes, leading to more severe illness and higher rates of healthcare utilization (13). However, the estimated benefit-to-cost ratio for SUD treatment is greater than 7:1, indicating that expanding access to and utilization of SUD treatment would yield significant benefits (6). This underscores the urgent need for innovative approaches and novel treatment options to address the ongoing mental health crisis associated with SUD (7).

The development of new approaches for treating SUD in recent years has reignited scientific interest in the use of psychedelics (9). Over the past decade, there has been a steady increase in the number of published review articles and clinical trial reports examining the potential therapeutic benefits of psychedelic compounds (14). Classic psychedelics include substances such as lysergic acid diethylamide (LSD), psilocybin, *N*,*N*-dimethyltryptamine (DMT), and mescaline. Non-classical psychedelics, such as 3,4-methylenedioxymethamphetamine (MDMA) and ketamine, also produce similar psychoactive effects (9). Among these, ketamine is particularly relevant as an *N*-methyl-d-aspartate receptor (NMDAR) antagonist that modulates glutamatergic neurotransmission, originally developed as a short-acting anesthetic (15).

Although dopaminergic pathways have traditionally been emphasized in the neurobiology of addiction (16), accumulating evidence suggests that glutamatergic dysregulation also plays a critical role in addiction. Notably, the glutamate projection from the prefrontal cortex to the accumbens core has been proposed as a final common pathway for initiating drug-seeking behavior (17). Therefore, glutamatergic dysfunction has been proposed as one of the neurobiological mechanisms underlying SUD and as a potential therapeutic target (18, 19). As an NMDAR antagonist, ketamine may influence addiction-related neuroadaptation through glutamatergic modulation and synaptic plasticity pathways (20). This mechanism may also be clinically relevant in SUD patients with comorbid affective symptoms (21), as depression and suicidal behaviors are common in this population (22–24). Previous reviews have suggested that ketamine’s rapid antidepressant and anti-suicidal effects are related to glutamatergic modulation and downstream mechanisms involving α-amino-3-hydroxy-5-methylisoxazole-4-propionic acid (AMPA) receptor activation, mammalian target of rapamycin (mTOR) signaling, brain-derived neurotrophic factor (BDNF), and glucose synthase kinase-3 (GSK3) pathways (25). At sub-anesthetic doses, ketamine may be beneficial for a range of psychiatric disorders, including depression and SUD (3). It has also been shown to alleviate withdrawal symptoms and reduce the risk of relapse (26). Although subanesthetic ketamine is generally well tolerated in controlled settings, its abuse and dependence liability remains a major safety concern (27). The drug’s euphoric, dissociative, and perceptual effects confer strong reinforcing properties that may promote repeated use (28). Chronic misuse has further been linked to cognitive deficits, emotional disturbances, urological complications, and potential excitotoxic neurotoxicity (27, 29–31). Emerging evidence suggests that combining ketamine with psychotherapy may enhance treatment outcomes (32). Psychotherapy may play a crucial role in prolonging the antidepressant and anti-addictive effects of ketamine (32). Preliminary evidence indicates that adjunctive psychotherapy can help sustain the positive effects of ketamine treatment for both SUD and depression (32).

Currently, there is no highly effective treatment for SUD, and clinical research on ketamine for SUD is relatively limited. There is a lack of systematic meta-analyses investigating the impact of ketamine on SUD. This study systematically reviews the literature with the aim of evaluating the efficacy of ketamine for SUD, as well as its safety and acceptability within the SUD population.

## Methods

### Search strategy

The study protocol has been registered in PROSPERO (CRD42024607116) and strictly adheres to the Preferred Reporting Items for Systematic reviews and Meta-Analyses (PRISMA) 2020 statement, ensuring methodological rigor and comprehensive transparency in systematic review reporting (33). A comprehensive literature search was performed across EMBASE, PubMed, Web of Science, and the Cochrane Library from their inception until May 5, 2026, with no restrictions on language. The search strategy was formulated using a combination of MeSH terms and keywords, incorporating terms such as substance use disorder, substance-related disorder, addiction, and ketamine. A detailed overview of the search strategy is provided in **Supplementary Table 1**. Two reviewers independently conducted the literature search and systematically screened titles and abstracts. Any discrepancies were addressed through full-text evaluation until a consensus was reached. In cases where consensus could not be attained, a third reviewer adjudicated the final decision.

### Selection procedure

#### Inclusion criteria and study selection

We included randomized controlled trials (RCTs) that met the following criteria:

1. Participant: Adults aged 18 years or older who either fulfilled the diagnostic criteria for any SUD as defined in any version of the Diagnostic and Statistical Manual of Mental Disorders (DSM) or the Structured Clinical Interview for DSM (SDID), or were identified as engaging in harmful substance use according to other established diagnostic criteria.
2. Intervention: Administration of ketamine, either as monotherapy or in conjunction with other therapeutic interventions, via any method of delivery. No restrictions were imposed on the dosing regimen of ketamine in the included studies.
3. Comparator: Studies with placebo-controlled groups or comparisons between different ketamine doses and/or administration frequencies.
4. Outcome: The primary efficacy outcome was the abstinence rate. The secondary outcomes included craving severity, the all-cause dropout rate (as an overall indicator of treatment acceptability), and the rate of adverse events (as a safety assessment).
5. Study design: Only RCTs were included.

#### Exclusion criteria

Studies were excluded if they met any of the following criteria:

1. included participants under 18 years of age;
2. studies of non-RCT designs, including non-randomized studies, animal studies, reviews, meta-analyses, and systematic reviews; and
3. absence of reported outcomes relevant to the primary objectives of this study.

### Data extraction

Two authors independently reviewed and extracted relevant data, including the following: 1) study characteristics: title, first author, publication date, country or region, and study design; 2) participant demographics and clinical characteristics: age, gender, ethnicity, diagnosis, and diagnostic criteria; and 3) methodological details: sample size, intervention type, route of administration, drug dosage, outcome measures, and assessment time points. 4) For the outcomes in the meta-analysis, we extracted means and standard deviations for continuous data, and frequencies and percentages for binary data. If key data were not fully reported, we attempted to extract information using the Engauge Digitizer software (34, 35) or to contact the corresponding author for additional details. We excluded from the analysis studies from which data could not be obtained through these methods.

### Risk-of-bias assessment

To assess the risk of bias in the included RCTs, two authors independently evaluated each study using the revised Cochrane risk-of-bias tool for randomized trials (36). The assessment encompassed seven domains, including random sequence generation (selection bias), allocation concealment (selection bias), blinding of participants and personnel (performance bias), blinding of outcome assessment (detection bias), incomplete outcome data (attrition bias), selective reporting (reporting bias), and other potential sources of bias. The risk of bias for each domain was classified as low, unclear, or high. A study was classified as having a low risk of bias if none of the assessed domains were rated as high risk, and no more than three domains were categorized as unclear risk. If a study had no domains rated as high risk but exhibited four or more domains classified as unclear risk, it was designated as having a moderate risk of bias. Any other scenario was considered to indicate a high risk of bias (37). Discrepancies between reviewers were resolved through discussion, and if consensus could not be reached, a third researcher was consulted for arbitration.

### GRADE assessment

We used the Grading of Recommendations, Assessment, Development, and Evaluation (GRADE) tool to assess the certainty of evidence for each outcome, classifying it as high, moderate, low, or very low (38). Evidence may be downgraded based on factors such as risk of bias, inconsistency, indirectness, imprecision, or publication bias.

### Statistical analysis

All statistical analyses were performed using Stata/MP 17.0. The M ± SD of continuous variables was extracted or calculated from the included studies. When different scales were used to assess the same outcome, the standardized mean difference (SMD) was employed as the effect size. For studies utilizing identical measurement methods, the weighted mean difference was used as the effect size. For binary variables (e.g., abstinence rate), the odds ratio (OR) was calculated as the effect size. Heterogeneity across studies was assessed using the I^2^ statistic, with the following interpretation (39): I^2^ = 0%–40%, likely insignificant heterogeneity; I^2^ = 30%–60%, possible moderate heterogeneity; I^2^ = 50%–90%, potentially substantial heterogeneity; and I^2^ = 75%–100%, considerable heterogeneity. Given the potential heterogeneity among studies, the random-effects model (40) was primarily employed. To explore potential sources of heterogeneity, subgroup analyses were performed based on the type of SUD, ketamine dosage and route of administration, frequency of administration, and intervention model, including ketamine without structured psychotherapy versus ketamine combined with structured psychotherapeutic interventions. Additionally, leave-one-out sensitivity analyses were performed to evaluate the robustness of the results. Publication bias was assessed through visual inspection of funnel plot symmetry and Egger’s regression test for statistical confirmation (41).

## Results

### Search results

**Figure 1:**
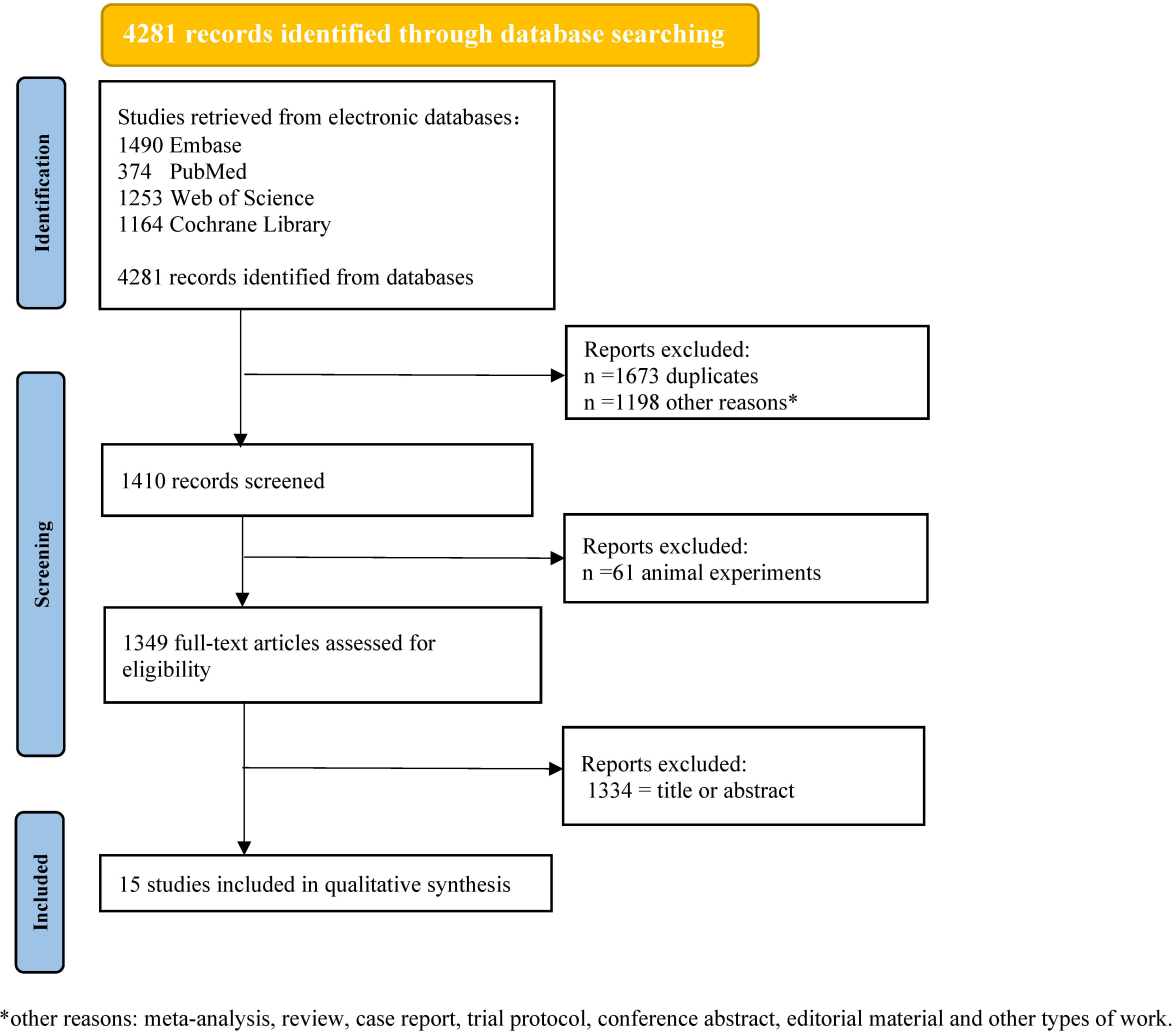
PRISMA flowchart of study selection process.

A total of 4,281 potentially relevant studies were identified through database searches and manual searches. Following independent screening by two reviewers, 15 studies met the eligibility criteria for inclusion in the systematic review (42–56). Detailed reasons for study exclusion are outlined in the PRISMA flow diagram (**Figure 1**).

### Main characteristics of included studies

A total of 15 RCTs involving 798 participants were included in this study, with 466 assigned to ketamine treatment. These trials comprised 11 double-blind trials (42–47, 49, 50, 52, 54, 55), three single-blind trials (48, 53, 56), and one open-label RCT (51). The participant populations varied across studies, including heroin dependence (two studies) (42, 44), opiate dependence (one study) (43), OUD (one study) (56), comorbid OUD and major depressive disorder (MDD) (one study) (54), comorbid MDD and opium use disorder (one study) (52), cocaine dependence (three studies) (45–47), AUD (four studies) (48–51), comorbid AUD and MDD (one study) (55), and TUD (one study) (53). Twelve studies administered ketamine via intravenous (IV) infusion (43, 45–55), and three utilized ketamine via intramuscular (IM) injection (42, 44, 56). Among the included studies, the Dakwar et al. study (2014) (45) and the Dakwar et al. study (2017) (46) were crossover double-blind trials, from which only pre-crossover phase outcomes were extracted for accuracy. Further details on these studies are provided in **Table 1**.

**Table 1:** Characteristics of the included studies.

Study	Type	Country	Diagnosis, diagnostic criteria	N	Age (M ± SD)	Gender (% male)	Race (%)	Routes	Dose	Measures	Time points
Krupitsky et al., 2002 (42)	RCT	Russia	Heroin dependence ICD-10/DSM-IV	70	22.30 (3.80)	55 (78.57%)	Not reported	IM	Ketamine (2.0 mg/kg), N = 35 Ketamine (0.20 mg/kg), N = 35	Abstinence rate, relapse rate, VASC	Post 1, 3, 6, 12, 18, 24 months
Jovaiša et al., 2006 (43)	RCT	Lithuania	Opiate dependence ICD-10/DSM-IV	50	23.09 (3.05)	43 (86.00%)	Not reported	IV	Ketamine (0.5 mg·kg^−1^·h^−1^), N = 22 Normal saline (0.5 mg·kg^−1^·h^−1^), N = 28	SOWS and OOWS Addiction Severity Index	Post 3 hours, 4 months
Krupitsky et al., 2007 (44)	RCT	Russia	Heroin dependence ICD-10/DSM-IV	59	22.60 (3.90)	49 (83.05%)	Not reported	IM	Single-session KPT (2.0 mg/kg), N = 27 Multiple-session KPT (2.0 mg/kg), N = 26	Abstinence rate, VASC	Post 12 months
Dakwar et al., 2014 (45)	RCT	United States	Cocaine dependence DSM-IV	8	47.50 (5.50)	7 (87.50%)	African American, 87.5% Hispanic, 12.5%	IV	Ketamine (0.41 mg/kg), N = 5 Lorazepam (2 mg), N = 3	URICA, VAS	Post 24 hours
Dakwar et al., 2017 (46)	RCT	United States	Cocaine dependence DSM-IV	20	48.60 (6.10)	11 (55.00%)	African American, 75% Hispanic, 20% White, 5%	IV	Ketamine (0.71 mg/kg), N = 10 Midazolam (0.025 mg/kg), N = 10	Cocaine self-administration Cocaine craving, VAS	Post 24 hours
Dakwar et al., 2019 (47)	RCT	United States	Cocaine dependence DSM-IV	55	47.00 (9.30)	41 (74.55%)	African American, 66.7% Caucasian, 13.7% Other or mixed, 19.6%	IV	Ketamine (0.5 mg/kg), N = 27 Midazolam (0.025 mg/kg), N = 28	Abstinence, time to relapse VAS	Post 2 weeks
Das et al., 2019 (48)	RCT	United Kingdom	AUD AUDIT score > 8	90	27.48 (8.11)	55 (61.11%)	Not reported	IV	RET + KET, N = 30 No RET + KET, N = 30 RET + PBO, N = 30	Drinking days	Post 7 days
Dakwar et al., 2020 (49)	RCT	United States	Alcohol dependence DSM-IV	40	53 (9.8)	19 (47.50%)	Asian, 5.4% Black/African American, 13.5% White, 70.3% Multiracial, 10.8%	IV	Ketamine (0.71 mg/kg, N = 17) Midazolam (0.025 mg/kg, N = 23)	Alcohol abstinence Heavy drinking days Time to relapse	Day 21, 6 months
Grabski et al., 2022 (50)	RCT	United Kingdom	AUD DSM-V/DSM-IV	96	44.10 (10.60)	61 (63.54%)	Not reported	IV	Ketamine (0.8 mg/kg) +PT (N = 24) Ketamine (0.8 mg/kg) +PE (N = 24) Placebo +PT (N = 23) Placebo +PE (N = 25)	Percentage of days abstinent Confirmed alcohol relapse ACQ	Post 6 months
Terasaki et al., 2022 (51)	RCT	United States	AUD DSM-V	44	45.11 (10.90)	35 (79.55%)	White/Caucasian, 56.8% Black/African American, 6.8% American Indian/Alaska Native, 15.9% Multiple/other, 20.5%	IV	KET (0.5 mg/kg), N = 13 IM NTX (380 mg), N = 14 LA, N = 17	All-cause hospital readmission rate	Post 30 days
Ahmadi et al., 2024 (52)	RCT	Iran	Comorbid MDD and opium use disorder DSM-V	66	40.65 (10.87)	65 (98.48%)	Not reported	IV	IV ketamine (0.5 mg/kg), N = 34 SL buprenorphine (16 mg), N = 32	BDI	Post 1 hour, 24 hours, 7 days
Chuang et al., 2025 (53)	RCT (pilot study)	United States	TUD Objective assessment	10	36.89 (12.61)	8 (80.00%)	White, 60% Black, 30% Other, 10%	IV	Ketamine (0.5 mg/kg), N = 6 Saline, N = 4	7-day cigarette diary QSU	Post 7 days
Mansoori et al., 2025 (54)	RCT	Iran	comorbid MDD and OUD DSM-V	64	40.76 (10.95)	59 (98.33%)	Not reported	IV	IV ketamine (0.5 mg/kg), N = 32 SL buprenorphine (16 mg), N = 32	HAMA Opioid Craving Scale	Post 2 hours, 24 hours, 7 days
Yoon et al., 2025 (55)	RCT	United States	comorbid MDD and AUD DSM-V	58	45.30 (12.80)	45 (77.59%)	White, 67.2% Black, 13.8% Hispanic or Latino, 13.8% Other, 5.20%	IV	IV ketamine (0.5 mg/kg) + IM naltrexone (380 mg), N = 20 IV ketamine plus IM saline, N = 19 IV midazolam (0.045 mg/kg) + IM saline, N = 19	Abstinence rate YCS	Post 4 weeks
Garland et al., 2026 (56)	RCT	USA	OUD	68	36.80 (9.20)	48 (70.60%)	White, 92.6% Other, 7.4%	IM	MORE + KAP (0.5–1.0 mg/kg), N = 34 MORE, N = 34	Instances of drug use VAS	Post 3 months

ACQ, Alcohol Craving Questionnaire; AUD, alcohol use disorders; AUDIT, Alcohol Use Disorders Identification Test; BDI, Beck Depression Inventory; DSM-IV, 4th edition of Diagnostic and Statistical Manual of Mental Disorders; DSM-V, 5th edition of Diagnostic and Statistical Manual of Mental Disorders; HAMA, Hamilton Anxiety Rating Scale; ICD-10, 10th Revision of International Classification of Diseases; IM, intramuscular; IV, intravenous; KAP, ketamine-assisted psychotherapy; KET, ketamine; KPT, ketamine psychotherapy; LA, linkage alone; MDD, major depressive disorder; MORE, Mindfulness-Oriented Recovery Enhancement; NTX, naltrexone; OOWS, Objective Opiate Withdrawal Scale; OUD, opioid use disorder; PBO, placebo; PE, psychoeducation; PT, psychotherapy; RCT, randomized controlled trial; QSU, Questionnaire of Smoking Urges; RET, retrieval; SOWS, Subjective Opiate Withdrawal Scale; TUD, tobacco use disorder; URICA, University of Rhode Island Change Assessment; VAS, Visual Analogue Scale; VASC, Visual Analog Scale of Craving; YCS, Yale Craving Scale.

### Risk-of-bias assessment

A detailed risk-of-bias assessment is presented in **Supplementary Figure 1**, while **Supplementary Figure 2** provides a risk-of-bias summary of the included studies. Overall, 11 studies were classified as low risk, and four were classified as high risk.

### Efficacy of ketamine in SUD

**Figure 2:**
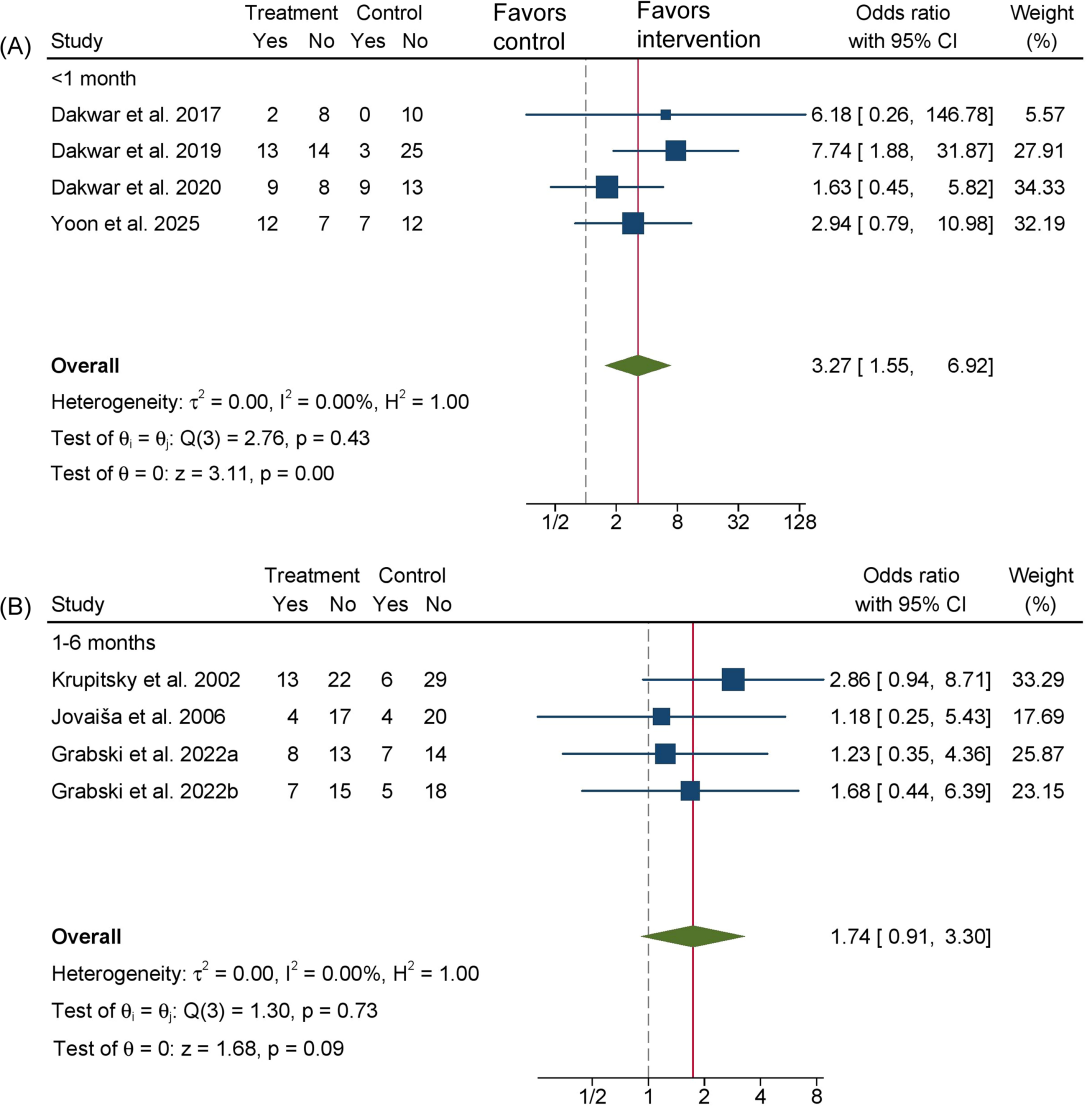
Meta-analysis comparing ketamine with the control group: abstinence rates.

Among the 15 RCTs identified, seven studies provided extractable data for the primary efficacy outcome, comprising 172 participants in the ketamine groups and 182 participants in the control groups. The other eight studies were excluded because they did not provide extractable binary abstinence data. Among the included studies, three investigated alcohol dependence, two cocaine dependence, and one each for heroin and opioid dependence. Abstinence was reported in all seven studies, although its operationalization varied according to the target substance and the design of the original trial. When explicitly defined, abstinence generally refers to no use of the target substance during a prespecified assessment period or follow-up interval, assessed by self-report, follow-up assessment, or timeline follow-back, with biochemical verification when available. Based on the extracted data, abstinence outcomes were not pooled into a single efficacy estimate because follow-up durations varied substantially across studies, ranging from 24 hours to 6 months. As acute abstinence does not necessarily indicate sustained recovery or relapse prevention, efficacy analyses were stratified into two follow-up intervals: <1 and 1–6 months. Four studies (46, 47, 49, 55) provided abstinence data for the <1-month interval, whereas three studies (42, 43, 50) reported abstinence outcomes for the 1–6-month interval. The meta-analysis showed that ketamine significantly improved abstinence rates in the <1-month analysis (OR = 3.27, 95% CI: 1.55–6.92, I^2^ = 0%, *p* < 0.01) (**Figure 2A**), whereas the effect in the 1–6-month analysis was favorable but not statistically significant (OR = 1.74, 95% CI: 0.91–3.30, I^2^ = 0%, *p* = 0.09) (**Figure 2B**).

For the secondary efficacy outcome of craving severity, seven comparisons from six studies provided extractable data for quantitative synthesis. Ketamine was not associated with a significant reduction in craving at <1 month (three studies; Hedges’s g = −0.09, 95% CI: −0.47 to 0.28, I^2^ = 0.00%, *p* = 0.62) (**Supplementary Figure 3A**), but was associated with greater craving reduction at 1–6 months (four comparisons; Hedges’s g = −0.38, 95% CI: −0.67 to −0.08, I^2^ = 16.84%, *p* = 0.01) (**Supplementary Figure 3B**).

### Subgroup analysis of efficacy

After stratification by follow-up duration, only four studies were included in the <1-month analysis and three studies in the 1–6-month analysis, limiting the feasibility of further subgroup analyses. The prespecified subgroup analysis by ketamine dose was not conducted because one subgroup was represented by only a single study, precluding meaningful formal subgroup comparison.

In the <1 month stratum, subgroup analyses were conducted according to intervention model and SUD type. By intervention model, the effect estimate was not statistically significant either for ketamine combined with structured psychotherapy (OR = 3.44, 95% CI: 0.75–15.84, I^2^ = 61.17%) or for ketamine without structured psychotherapy (OR = 3.28, 95% CI: 0.97–11.08, I^2^ = 0%), with no significant subgroup difference observed (*p* = 0.96) (**Supplementary Figure 4**). By SUD type, ketamine was significantly associated with abstinence in cocaine use disorder (OR = 7.45, 95% CI: 2.05–27.14, I^2^ = 0%), but not in alcohol use disorder (OR = 2.16, 95% CI: 0.87–5.42, I^2^ = 0%); however, the between-subgroup difference was not significant (*p* = 0.13) (**Supplementary Figure 5**). In the 1–6 months stratum, subgroup analysis by administration frequency showed no statistically significant effect for either single administration (OR = 2.10, 95% CI: 0.85–5.17, I^2^ = 0%) or multiple administration (OR = 1.43, 95% CI: 0.57–3.57, I^2^ = 0%), with no significant subgroup difference (*p* = 0.56) (**Supplementary Figure 6**). Exploratory subgroup analysis of craving outcomes at 1–6 months showed that the pooled effect estimates were similar in magnitude for IM ketamine (Hedges’s g = −0.38, 95% CI: −0.84 to 0.08, I^2^ = 47.97%) and IV ketamine (Hedges’s g = −0.38, 95% CI: −0.94 to 0.18, I^2^ = 40.66%). However, neither subgroup reached statistical significance, and there was no evidence of a between-subgroup difference (*p* = 0.99) (**Supplementary Figure 7**). Overall, these subgroup analyses were exploratory and should be interpreted cautiously given the limited number of studies and imprecise effect estimates.

### Dropout rates

Twelve studies involving 580 participants provided dropout data for any reason. The meta-analysis results indicated that there was no statistically significant difference in dropout rates between the ketamine and control groups (OR = 0.74, 95% CI: 0.46–1.21, I^2^ = 0%, *p* = 0.23) (**Supplementary Figure 8**).

### Adverse events

We conducted a meta-analysis of 12 studies that reported adverse events. The findings indicated no significant difference between ketamine and placebo in the incidence of serious adverse events (OR = 0.71, 95% CI: 0.24–2.04, I^2^ = 0%, *p* = 0.52) (**Supplementary Figure 9**) or the rate of other (excluding serious) adverse events (OR = 0.87, 95% CI: 0.50–1.50, I^2^ = 0%, *p* = 0.61) (**Supplementary Figure 10**).

### Sensitivity analyses

We performed leave-one-out analyses to assess the influence of individual studies on the pooled estimates for abstinence, all-cause dropout, and adverse events. The results showed that all findings were robust (**Supplementary Figures 11**-**S14**).

### Publication bias

We assessed the potential for publication bias using funnel plots and Egger’s regression test. For abstinence rates, all-cause dropout rates, and the rate of serious and non-serious adverse events, neither the funnel plots nor Egger’s test showed significant evidence of bias (*p* > 0.05) (**Supplementary Figures 15**-**S18**). However, the limited number of studies constrains our ability to rule out publication bias.

### Quality of evidence

The GRADE assessment indicated moderate-certainty evidence supporting an improvement in abstinence rates within 1 month after ketamine treatment. However, the evidence for abstinence at 1–6 months was of low certainty and did not demonstrate a clear sustained benefit. Thus, the long-term effect of ketamine on abstinence remains uncertain. For all-cause dropout and adverse events, no significant differences were observed between the ketamine and control groups, but the certainty of evidence was very low because of very serious risk of bias and serious imprecision. Therefore, current evidence remains insufficient to draw firm conclusions regarding the acceptability and safety of ketamine in patients with SUD. A summary of the GRADE evidence is presented in **Table 2**.

**Table 2:** Summary of GRADE evidence.

Outcome	No. of participants	Certainty in evidence	Detailed reasons
Abstinence rate <1 month 1–6 months	N = 152 (4 RCTs) N = 202 (3 RCTs)	Moderate Low	Downgraded for serious inconsistency Downgraded for serious imprecision and serious inconsistency
All-cause dropout rate	N = 580 (12 RCTs)	Very low	Downgraded for very serious RoB and serious imprecision
Adverse events rate	N = 594 (12 RCTs)	Very low	Downgraded for very serious RoB and serious imprecision

GRADE, Grading of Recommendations, Assessment, Development, and Evaluation; RCT, randomized controlled trial; RoB, risk of bias.

Very low = any estimate of effect is very uncertain; Low = further research is very likely to have an important impact on our confidence in the estimate of effect and is likely to change the estimate; Moderate = further research is likely to have an important impact on our confidence in the estimate of effect and may change the estimate.

## Discussion

In this study, we synthesized existing evidence to evaluate the potential of ketamine in treating SUD. The included studies indicate that ketamine significantly improves abstinence rates compared to control groups, despite differences in study design, populations, and administration routes. Early studies on OUD have suggested varying effects of ketamine on abstinence rates. Krupitsky et al. (42) found that ketamine significantly reduced heroin cravings and increased abstinence rates, while Jovaiša et al. (43) reported that while ketamine notably alleviated acute withdrawal symptoms early on, it had limited effects on long-term abstinence. Regarding cocaine use disorder, Dakwar et al. (47) demonstrated that a single ketamine infusion combined with mindfulness-based behavioral interventions significantly improved abstinence rates, reduced relapse risk, and decreased cravings, with sustained benefits observed at 6 months. Additionally, studies on AUD have yielded mixed results. Dakwar et al. (49) and Grabski et al. (50) found that ketamine increased alcohol abstinence rates, whereas Yoon et al. (55) reported no significant differences in abstinence rates, alcohol cravings, or daily alcohol consumption compared to the control group.

Ketamine was associated with improved abstinence at <1 month, but this effect was not sustained at >1 month. Conversely, ketamine showed no significant effect on craving severity at <1 month, but was associated with a modest reduction at >1 month. This pattern may reflect differences between abstinence and craving as clinical endpoints: abstinence is a behavioral outcome, whereas craving is a subjective motivational state that may change over a different time course. Overall, current evidence suggests that ketamine may benefit selected SUD-related outcomes, although the findings remain preliminary. In addition to these efficacy findings, ketamine was not associated with a clear increase in all-cause dropout or adverse event rates compared with control conditions. However, these findings should be interpreted cautiously, as the available evidence remains limited and insufficient to draw firm conclusions regarding acceptability or safety. Although the I^2^ value for the primary outcome was 0%, the absence of statistically detected heterogeneity should not be interpreted as evidence of true clinical or methodological homogeneity. Given that only seven studies contributed to the primary abstinence analysis and only six studies contributed to the craving analysis, with relatively small sample sizes for both outcomes, the assessment of heterogeneity was likely underpowered, and a false-negative finding for between-study heterogeneity cannot be ruled out. This is particularly important given the substantial differences across trials in SUD subtype, route of administration, dose, control condition, follow-up duration, and whether ketamine was administered as monotherapy or adjunctive to psychotherapeutic interventions. Therefore, the pooled estimate should be interpreted as a preliminary signal requiring confirmation, rather than as definitive evidence of a uniform treatment effect across SUD populations or intervention models.

Exploratory subgroup analyses did not provide robust evidence that ketamine efficacy differed by SUD type, intervention model, administration frequency, or route of administration. Subgroup findings should be interpreted cautiously. In the <1-month stratum, the significant effect in cocaine use disorder was not supported by a significant between-subgroup difference, limiting any inference of substance specificity. For AUD, although the pooled estimate was not statistically significant (OR = 2.16, 95% CI: 0.87–5.42), the wide confidence interval was compatible with a potentially meaningful benefit, suggesting limited power rather than definitive absence of effect. Larger trials are needed to clarify these signals. Likewise, the null comparison between single and multiple administrations in the 1–6 months stratum should not be interpreted as evidence that dosing frequency is unimportant, but rather as a reflection of limited power and statistical imprecision. This interpretation is consistent with that of Krupitsky et al. (44), who reported that three sessions of ketamine-assisted psychotherapy were more effective in promoting long-term abstinence in heroin-dependent individuals than a single session, with 50% of the three-session group maintaining abstinence after 1 year, compared to only 22.2% in the single-session group. Interpretation of these subgroup findings is further complicated by the frequent co-administration of psychotherapeutic interventions, which makes it difficult to disentangle the independent pharmacological effect of ketamine from potential synergistic treatment effects. Overall, these subgroup analyses were exploratory and should be interpreted with caution.

In the 15 studies with available data, ketamine did not significantly increase the all-cause dropout rate compared to the control group, suggesting that its acceptability is comparable to that of the control group. However, the wide confidence intervals limit the interpretability of this conclusion. No significant differences were found in the incidence of serious and non-serious adverse events between the ketamine and control groups, indicating that ketamine does not signal increased harm. Nevertheless, given the broad confidence intervals, the safety of ketamine has not been fully established. All included studies were conducted in controlled environments, which prevents an assessment of the risks associated with ketamine use in other settings. Moreover, comparable overall adverse event rates do not exclude ketamine-specific safety concerns. Ketamine may induce psychedelic-like experiences and subjective effects such as dizziness, out-of-body experiences, altered reality perception, and altered time perception (42, 50). Compared with midazolam, ketamine was associated with significantly higher acute dissociation ratings; however, all psychoactive effects resolved within 30 minutes after infusion, and no persistent dissociation was observed (47). In addition, ketamine may induce transient cardiovascular responses, which may explain why uncontrolled hypertension was listed as a key exclusion criterion for participants receiving ketamine in some studies (50, 53). Krupitsky et al. reported an acute increase in systolic and especially diastolic blood pressure of approximately 20%–30% following ketamine treatment (42, 44). In addition to these acute effects, concerns about ketamine misuse and the long-term risks associated with illicit use, including urological and cognitive effects, suggest that structured clinical protocols in controlled settings appear to effectively mitigate these safety concerns (20, 57).

Our study has several inherent limitations that should be considered when interpreting the results. First, although the body of research on ketamine for depression is relatively extensive, RCTs on ketamine for SUD are limited. Although 15 RCTs were included in the systematic review, only seven trials provided extractable binary abstinence data for the primary meta-analysis. The remaining studies were retained in the qualitative synthesis but were not pooled because their outcomes were not compatible with abstinence-rate meta-analysis. Accordingly, the overall efficacy estimate should be interpreted with caution, as it reflects only the quantitatively synthesizable subset of the evidence base. In particular, heroin and opioid-related disorders were each represented by only a single study in the abstinence analysis. Therefore, these categories were not considered suitable for formal subgroup analyses or reliable substance-specific inference and were instead summarized descriptively. Additionally, there were significant variations across studies in terms of SUD subtypes, intervention protocols, combinations with psychotherapy, control groups, and follow-up durations. Second, some trials combined ketamine with psychotherapy, making it difficult to differentiate the independent effects of ketamine from the synergistic effects of combined treatments. Direct comparisons between ketamine monotherapy and ketamine-assisted psychotherapy remain limited. Third, the results of exploratory subgroup analyses, such as those for IM administration or anesthetic doses, were based on single studies and should be considered preliminary rather than definitive. Finally, a key limitation of the included trials is the potential for functional unblinding. Ketamine produces distinctive dissociative and psychedelic-like subjective effects, whereas the control conditions used across studies may not have adequately mimicked these experiences. Therefore, participants may have been able to infer their treatment allocation despite formal blinding. This may have introduced expectancy effects and performance bias, particularly for outcomes such as abstinence, craving, relapse, and motivation to change. As a result, the observed efficacy of ketamine may be inflated and should not be interpreted as a purely pharmacological effect. Future research should prioritize adequately powered and methodologically rigorous RCTs with robust strategies to minimize and evaluate functional unblinding in ketamine interventions for SUD. Standardized outcome measures, including abstinence, craving, relapse, and functional recovery, are needed to improve comparability across studies. Given the rapidly evolving evidence base, longer and more systematic follow-up is essential to assess durability of response, relapse prevention, misuse potential, and delayed adverse effects. Future trials should also clarify the independent contribution of ketamine versus ketamine-assisted psychotherapy, optimize dosing and administration routes, compare racemic ketamine with its enantiomers, and implement robust functional blinding to minimize potential biases.

## Conclusions

This study provides preliminary evidence regarding the efficacy, acceptability, and safety of ketamine for the treatment of SUD. The findings suggested that ketamine may improve abstinence rates within 1 month in individuals with SUD. Although the available evidence suggested a signal of efficacy for specific subgroups, these findings should be interpreted with caution and require confirmation in high-quality, well-blinded randomized controlled trials before any clinical recommendations can be made. No significant differences were observed between ketamine and placebo with respect to abstinence rates at 1–6 months, acceptability, or adverse events. However, given the limited sample sizes, heterogeneity across studies, and the low certainty of evidence for these outcomes, current evidence remains insufficient to establish the sustained efficacy of ketamine beyond 1 month or to confirm its acceptability and safety in this population. Overall, the current evidence base remains limited, and the conclusions of this study should be considered preliminary. Further high-quality studies are needed to validate and extend these findings.

## Data Availability

The original contributions presented in the study are included in the article/supplementary material. Further inquiries can be directed to the corresponding author.

## Glossary

SUD: substance use disorder
RCTs: randomized controlled trials
AUD: alcohol use disorder
MAT: medication-assisted treatment
FDA: the U.S. Food and Drug Administration
TUD: tobacco use disorder
NRTs: nicotine replacement therapies
OUD: opioid use disorder
AmUD: amphetamine use disorder
CaUD: cannabis use disorder
CoUD: cocaine use disorder
LSD: lysergic acid diethylamide
MDMA: 3,4-methylenedioxymethamphetamine
NMDAR: *N*-methyl-{{sc}}d{{/sc}}-aspartate receptor
AMPA: α-amino-3-hydroxy-5-methylisoxazole-4-propionic acid
mTOR: mammalian target of rapamycin
BDNF: brain-derived neurotrophic factor
GSK3: glucose synthase kinase-3
GRADE: Grading of Recommendations, Assessment, Development, and Evaluation
SMD: standardized mean difference
WMD: weighted mean difference
OR: odds ratio
OUD: opium use disorder
MDD: major depressive disorder
IV: intravenous
IM: intramuscular
URICA: University of Rhode Island Change Assessment
BDI: the Beck Depression Inventory
TRD: treatment-resistant depression
AUDIT: Alcohol Use Disorders Identification Test
DSM-IV: 4th edition of Diagnostic and Statistical Manual of Mental Disorders
DSM-V: 5th edition of Diagnostic and Statistical Manual of Mental Disorders
ICD-10: 10th Revision of International Classification of Diseases
KET: ketamine
KPT: ketamine psychotherapy
LA: linkage alone
MBI: mindfulness-based intervention
NTX: naltrexone
OOWS: Objective Opiate Withdrawal Scale
PE: psychoeducation
PT: psychotherapy
RET: retrieval
SOWS: Subjective Opiate Withdrawal Scale
