# Supplementary material for: Ketamine for substance use disorders: a systematic review and meta-analysis
Source: Front Psychiatry. 2026 Jul 17;17:1835709. doi: 10.3389/fpsyt.2026.1835709 (PMC13426052; doi:10.3389/fpsyt.2026.1835709)

**Supplementary material**

**Ketamine for substance use disorders: A systematic review and meta-analysis**

**Summary**

**Supplementary Table S1.** Database search strategy for meta-analysis.

**Supplementary Figure S1.** Risk of bias assessment of the included studies.

**Supplementary Figure S2.** Bias risk summary of the included studies.

**Supplementary Figure S3**. Time-stratified meta-analysis of craving severity.

**Supplementary Figure S4.** Subgroup analysis of abstinence rates by intervention model in the <1 month stratum.

**Supplementary Figure S5.** Subgroup analysis of abstinence rates by substance use disorder type in the <1 month stratum.

**Supplementary Figure S6**. Subgroup analysis of abstinence rates by administration frequency in the 1–6 months stratum.

**Supplementary Figure S7.** Subgroup analysis of craving severity by route of administration in the 1–6 months stratum.

**Supplementary Figure** **S8**. Meta-analysis of ketamine versus the control group: Dropout rate.

**Supplementary Figure** **S9**. Meta-analysis of ketamine versus the control group: Serious adverse events.

**Supplementary Figure** **S10**. Meta-analysis of ketamine versus the control group: Other adverse events.

**Supplementary Figure** **S11**. Sensitivity analysis of abstinence rates.

**Supplementary Figure** **S12**. Sensitivity analysis of dropout rates.

**Supplementary Figure** **S13**. Sensitivity analysis of Serious adverse events.

**Supplementary Figure** **S14**. Sensitivity analysis of Other adverse events.

**Supplementary Figure** **S15.** Funnel plot for assessing the publication bias for abstinence rates.

**Supplementary Figure** **S16.** Funnel plot for assessing the publication bias for dropout rates.

**Supplementary Figure** **S17.** Funnel plot for assessing the publication bias for serious adverse events.

**Supplementary Figure** **S18.** Funnel plot for assessing the publication bias for other adverse events.

**Supplementary Table S1.** Database search strategy for meta-analysis.

PubMed

| No. | Query | Results |
| --- | --- | --- |
| 1 | ((Substance Related Disorder[MeSH Terms]) OR ("substance use disorder")) OR (craving[Title/Abstract]) OR (withdrawal[Title/Abstract]) OR (addiction[Title/Abstract]) OR (alcohol[Title/Abstract]) OR (opioids[Title/Abstract]) OR (tobacco[Title/Abstract]) OR (cocaine[Title/Abstract]) OR (nicotine[Title/Abstract]) | 910,539 |
| 2 | Ketamine[MeSH Terms] OR Ketamine[Title/Abstract] | 29,000 |
| 3 | ((((((Randomized Controlled Trial[Publication Type]) OR (controlled study[Title/Abstract])) OR (Double-Blind[Title/Abstract])) OR (Single-Blind[Title/Abstract])) OR (Placebo[Title/Abstract])) OR (Randomized Controlled Trial[Title/Abstract])) | 897,631 |
| 4 | 1 AND 2 AND 3 | 374 |

Cochrane Library

| No. | Query | Results |
| --- | --- | --- |
| 1 | (Substance Related Disorder):ti,ab,kw OR (Substance use disorder):ti,ab,kw OR (craving):ti,ab,kw OR (withdrawal):ti,ab,kw OR (addiction) :ti,ab,kw OR (alcohol) :ti,ab,kw OR (opioid) :ti,ab,kw OR (tobacco) :ti,ab,kw OR (cocaine) :ti,ab,kw OR (nicotine) :ti,ab,kw (Word variations have been searched) | 135141 |
| 2 | MeSH descriptor: [Substance Related Disorder] explode all trees | 22205 |
| 3 | 1 OR 2 | 139137 |
| 4 | (Ketamine):ti,ab,kw | 8856 |
| 5 | MeSH descriptor: [Ketamine] explode all trees | 3512 |
| 6 | 4 OR 5 | 8856 |
| 7 | (Randomized controlled trial):pt OR (Double-Blind):ti,ab,kw OR (Single-Blind):ti,ab,kw OR (Placebo):ti,ab,kw OR (Randomized Controlled Trial):ti,ab,kw | 1168302 |
| 8 | 3 AND 6 AND 7 | 1164 |

Embase

| No. | Query | Results |
| --- | --- | --- |
| 1 | 'drug dependence'/exp OR 'drug dependence':ti,ab,kw OR 'substance related disorder':ti,ab,kw OR sud:ti,ab,kw OR craving:ti,ab,kw OR withdrawal:ti,ab,kw OR addiction:ti,ab,kw OR alcohol:ti,ab,kw OR opioid:ti,ab,kw OR tobacco:ti,ab,kw OR cocaine:ti,ab,kw OR nicotine:ti,ab,kw | 1,247,184 |
| 2 | 'randomized controlled trial'/de OR 'randomized controlled trial':it OR 'controlled study':ti,ab,kw OR 'double blind':ti,ab,kw OR 'single blind':ti,ab,kw OR placebo:ti,ab,kw | 1,514,020 |
| 3 | 'ketamine'/exp OR 'ketamine':ti,ab,kw | 87,689 |
| 4 | 1 AND 2 AND 3 | 1490 |

Web of Science

| No. | Query | Results |
| --- | --- | --- |
| 1 | TS=(Ketamine) | 27,455 |
| 2 | ((((((((((TS=(Substance Related Disorder)) OR TS=(substance use disorder)) OR TS=(craving)) OR TS=(withdrawal)) OR TS=(addiction)) OR TS=(alcohol)) OR TS=(opioid)) OR TS=(tobacco)) OR TS=(cocaine)) OR TS=(nicotine)) | 1,076,277 |
| 3 | ((((TS=(randomized controlled trial)) OR TS=(Double-Blind)) OR TS=(Single-Blind)) OR TS=(Placebo))OR TS=(random*) | 2,769,057 |
| 4 | 1 AND 2 AND 3 | 1,253 |

**Supplementary Figure S1.** Risk of bias assessment of the included studies.


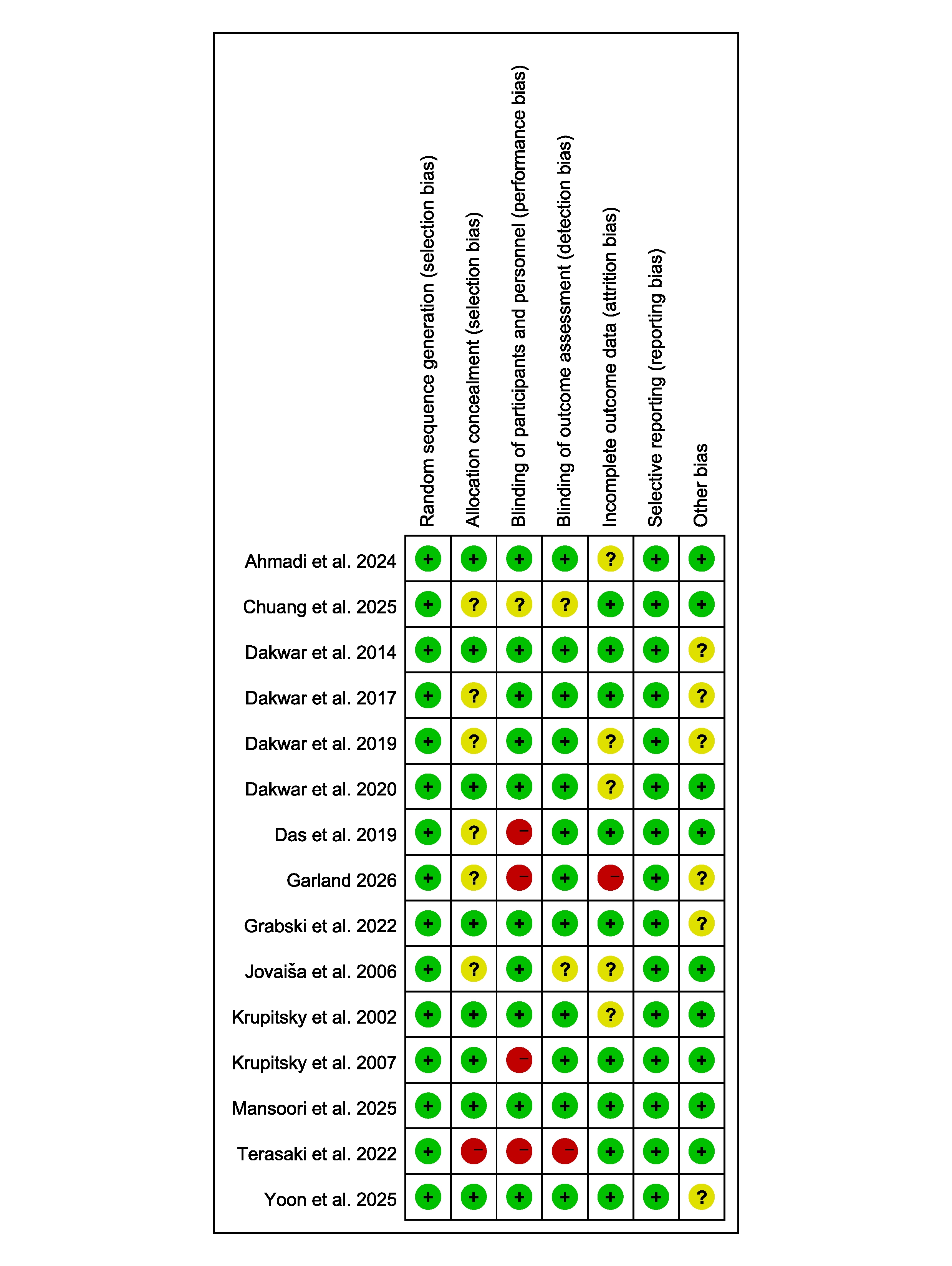


**Supplementary Figure S2.** Bias risk summary of the included studies.


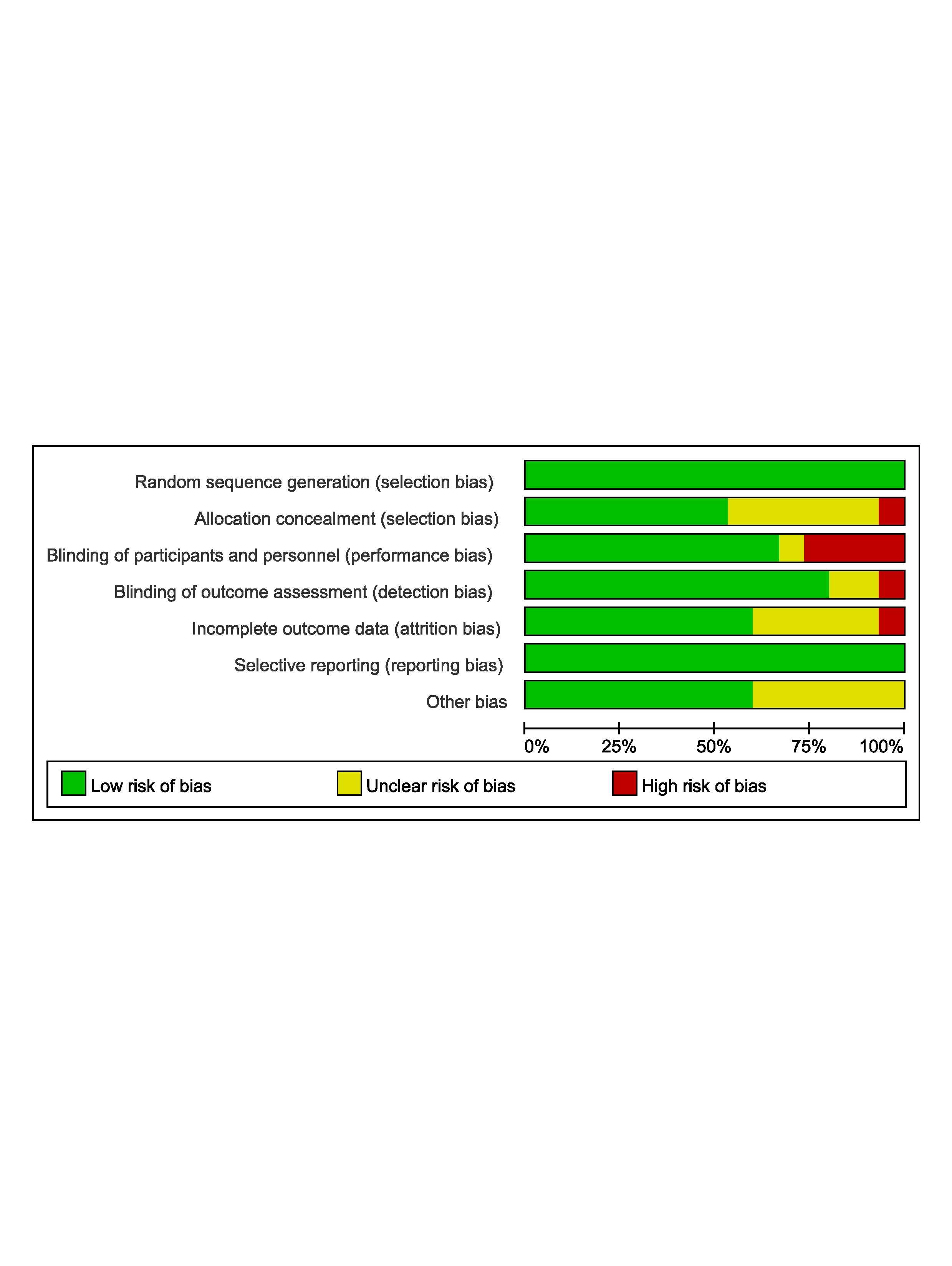


**Supplementary Figure S3**. Time-stratified meta-analysis of craving severity.





**Supplementary Figure S4.** Subgroup analysis of abstinence rates by intervention model in the <1 month stratum.


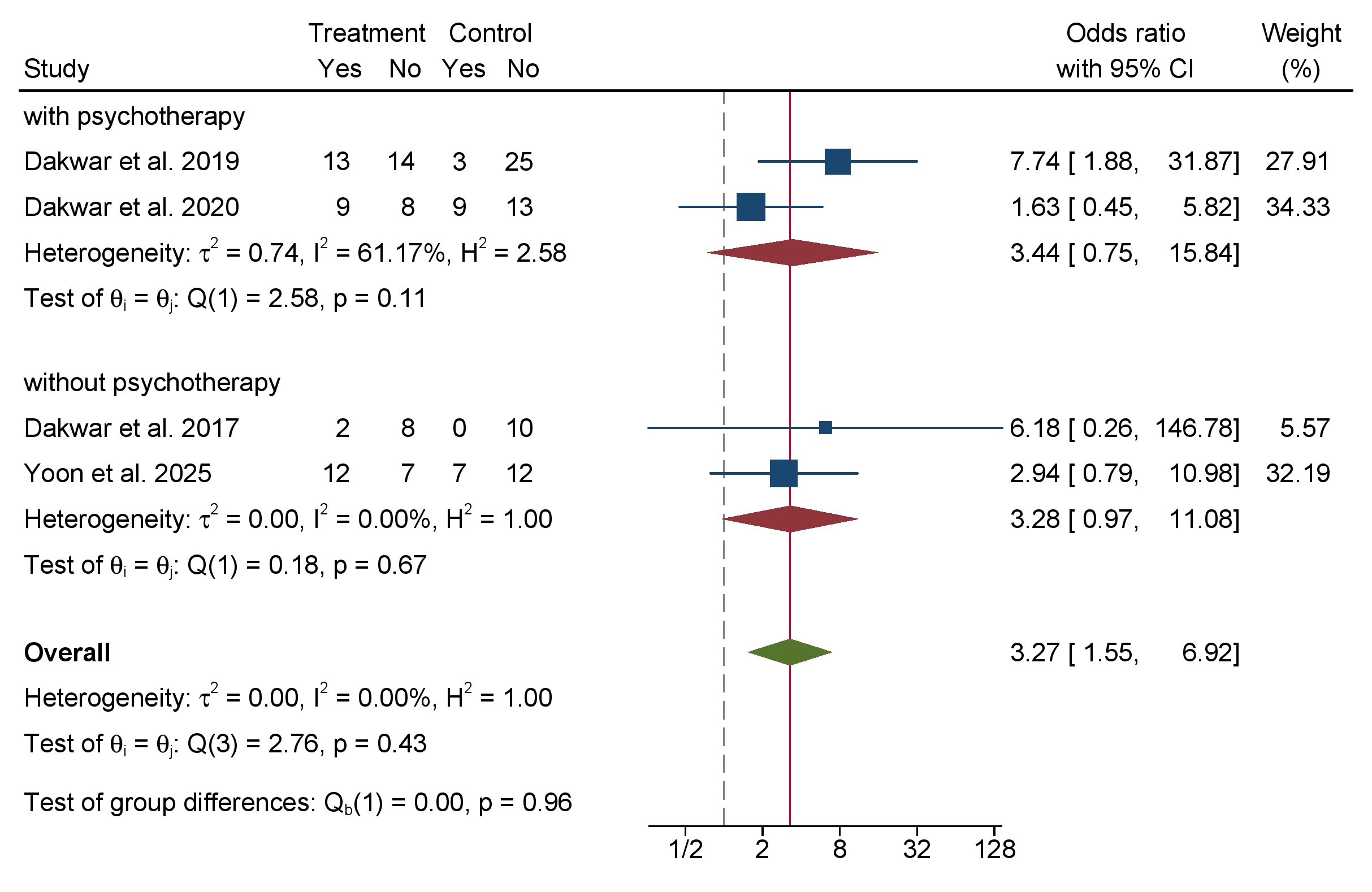


**Supplementary Figure S5.** Subgroup analysis of abstinence rates by substance use disorder type in the <1 month stratum.


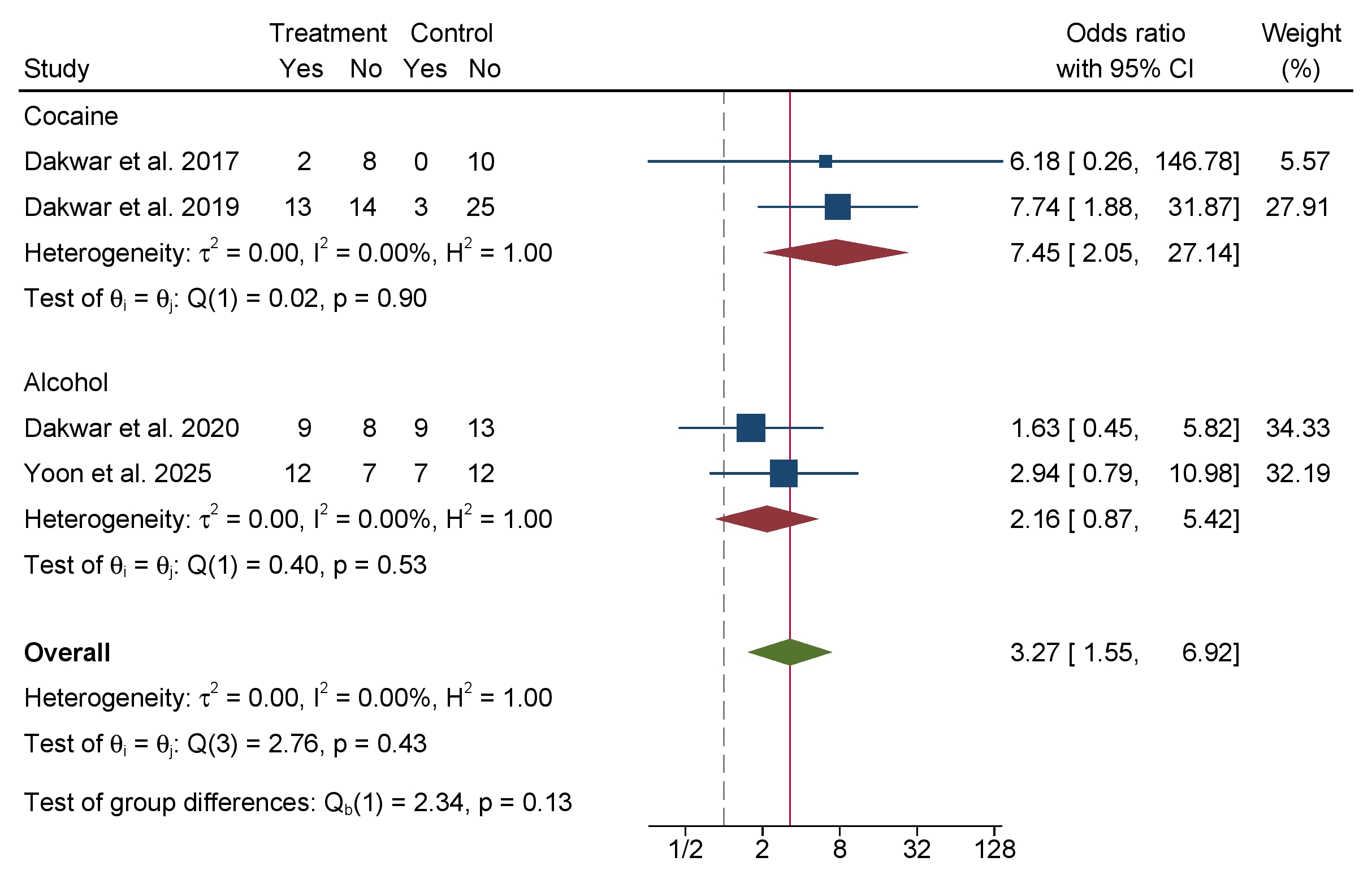


**Supplementary Figure S6**. Subgroup analysis of abstinence rates by administration frequency in the 1–6 months stratum.


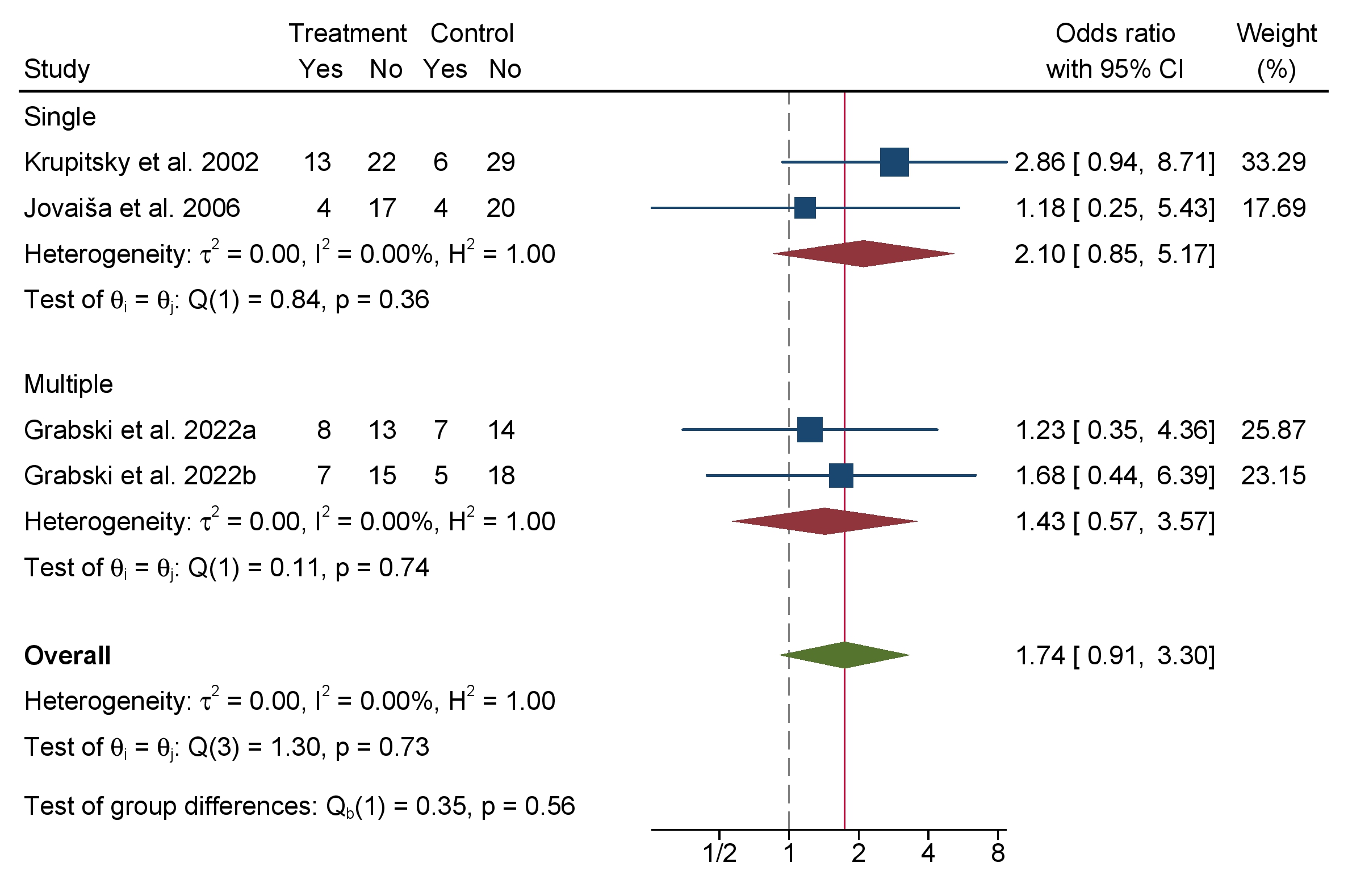


**Supplementary Figure S7.** Subgroup analysis of craving severity by route of administration in the 1–6 months stratum.


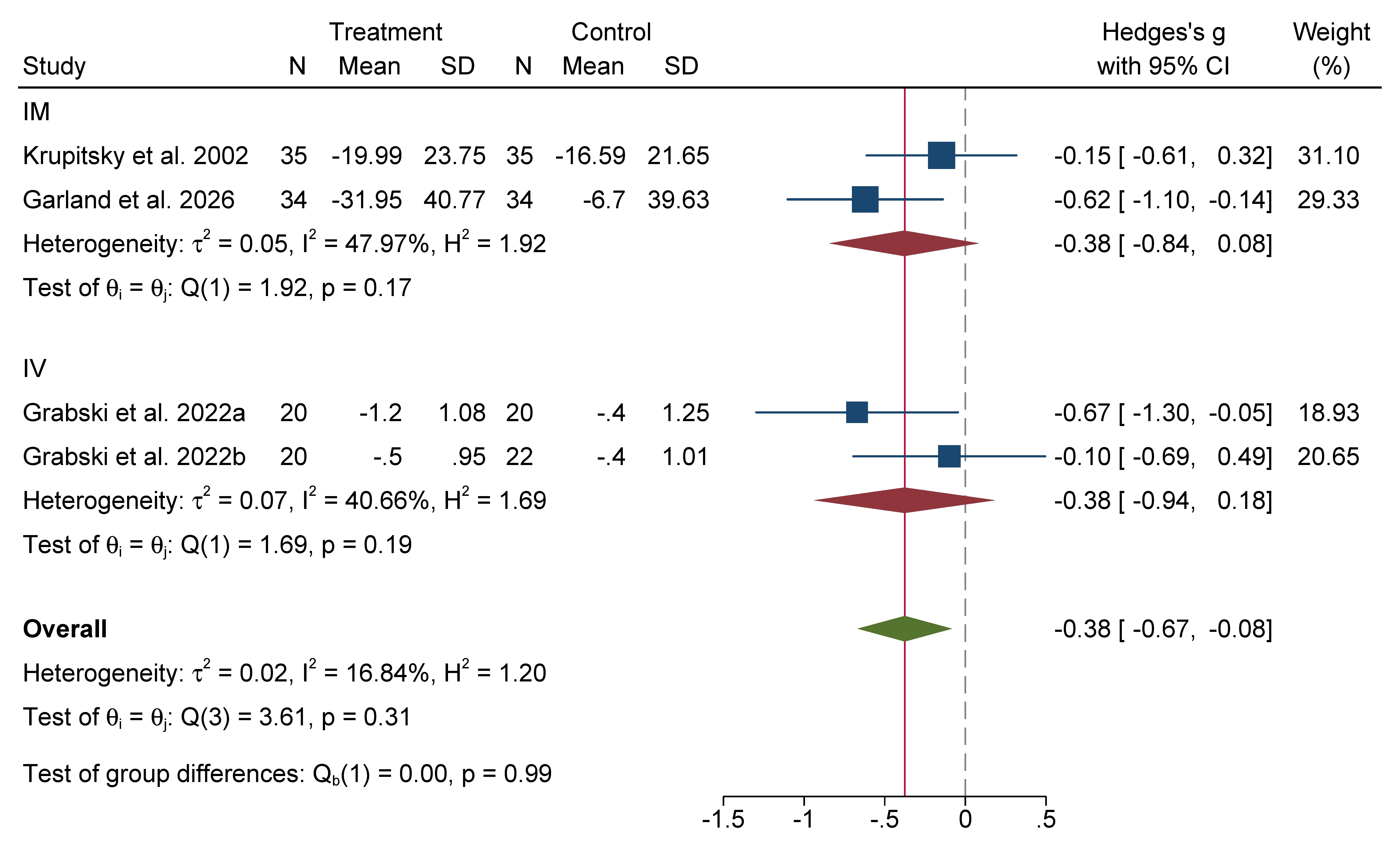


**Supplementary Figure** **S8**. Meta-analysis of ketamine versus the control group: Dropout rate.


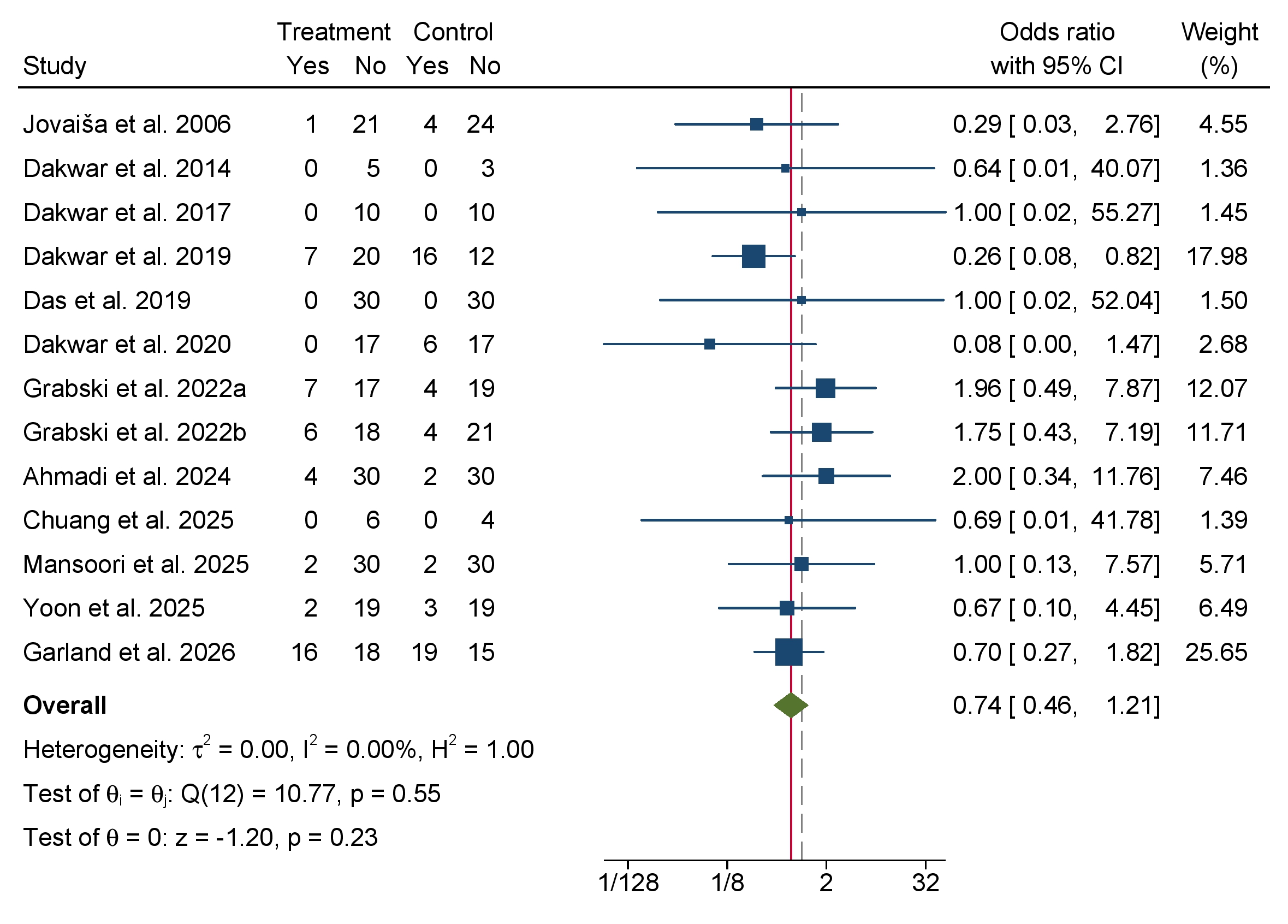


**Supplementary Figure** **S9**. Meta-analysis of ketamine versus the control group: Serious adverse events.


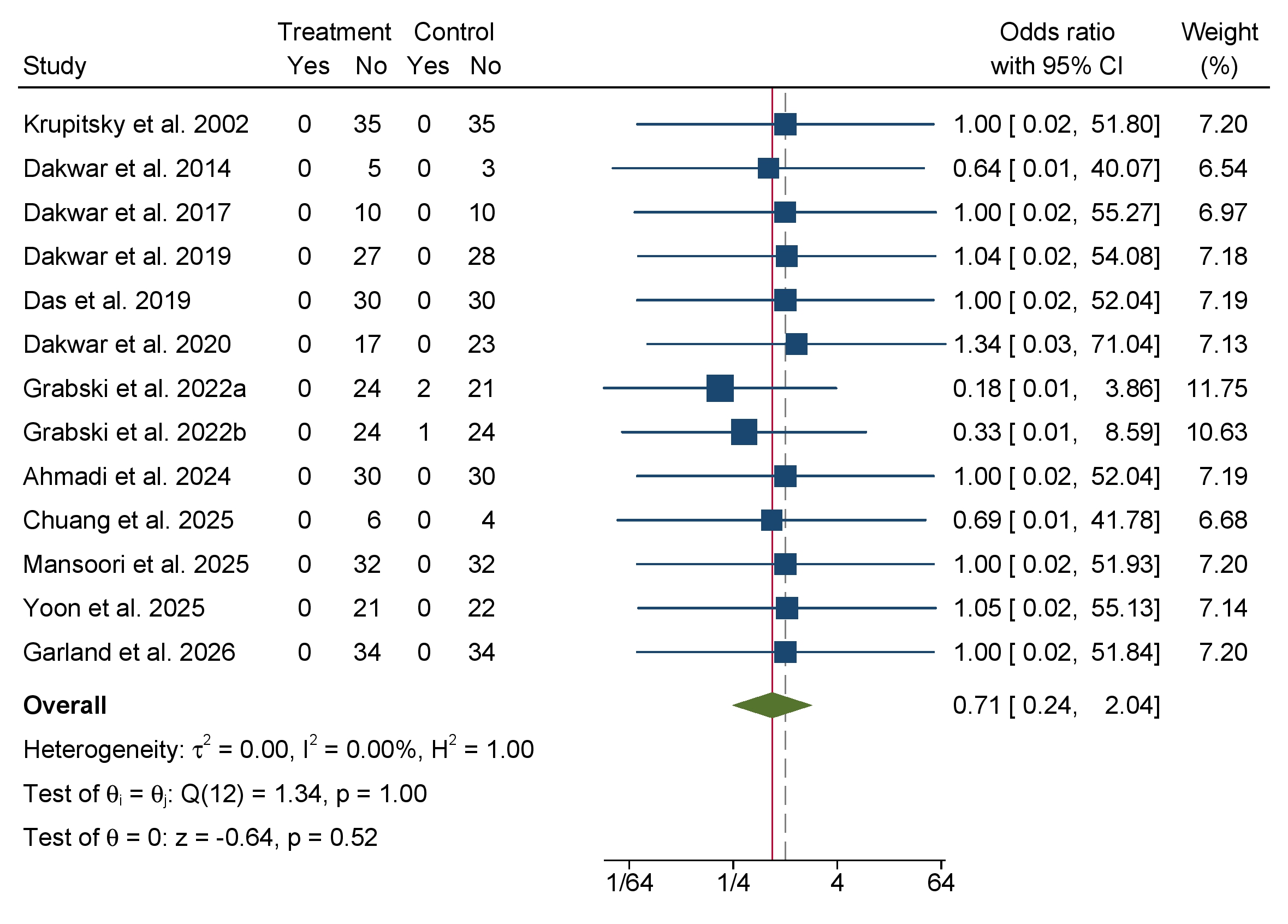


**Supplementary Figure** **S10**. Meta-analysis of ketamine versus the control group: Other adverse events.


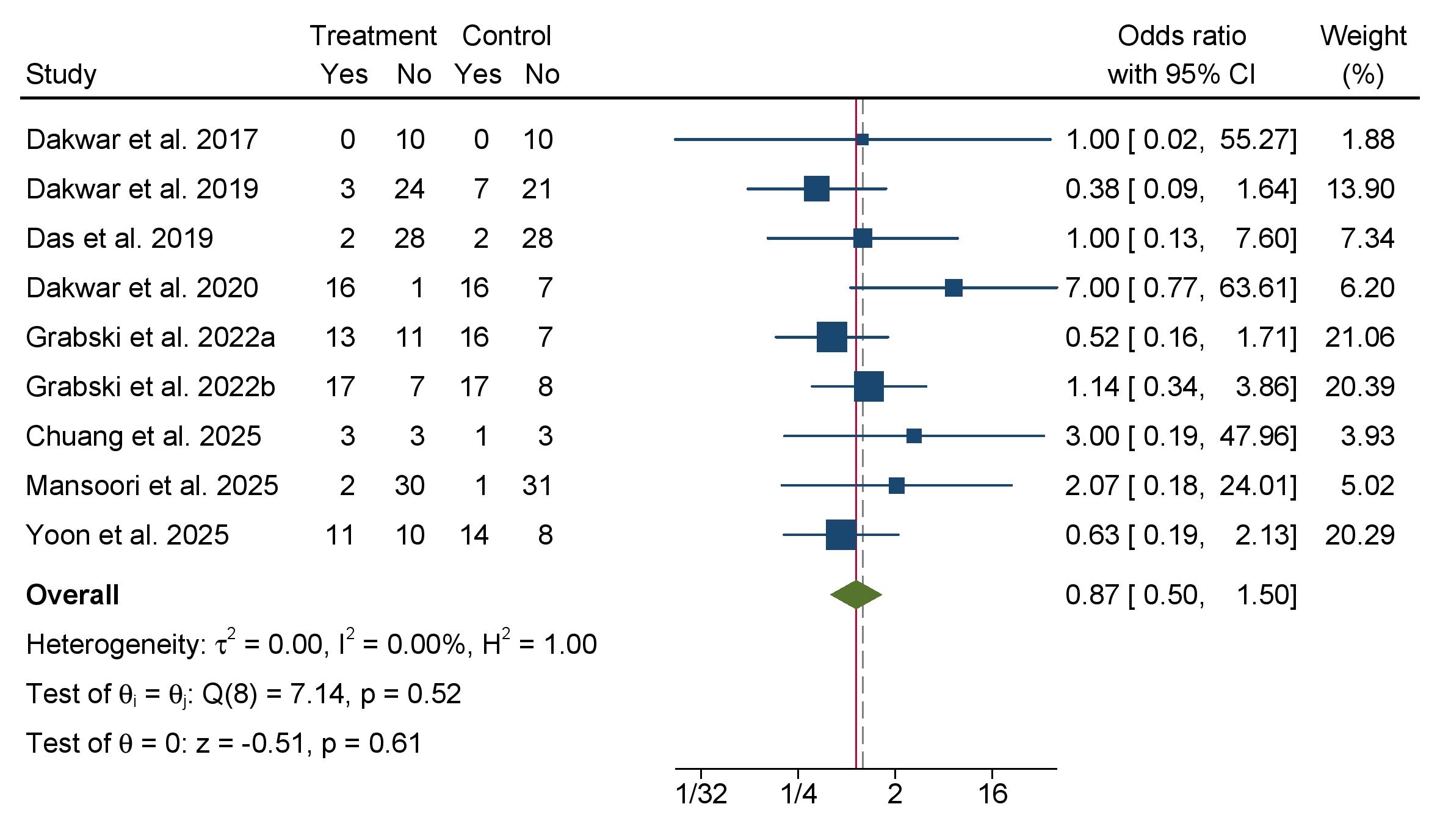


**Supplementary Figure** **S11**. Sensitivity analysis of abstinence rates.


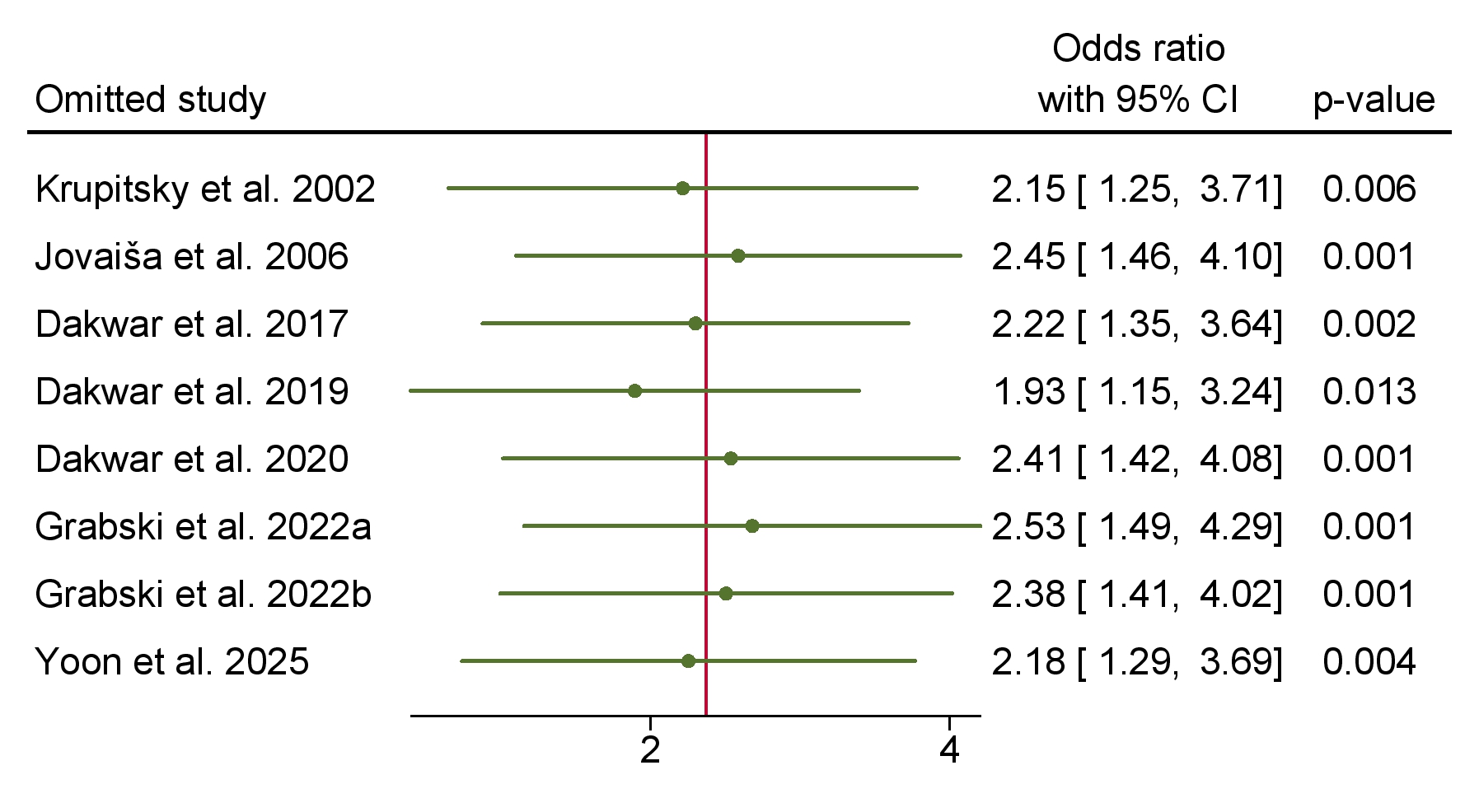


**Supplementary Figure** **S12**. Sensitivity analysis of dropout rates.


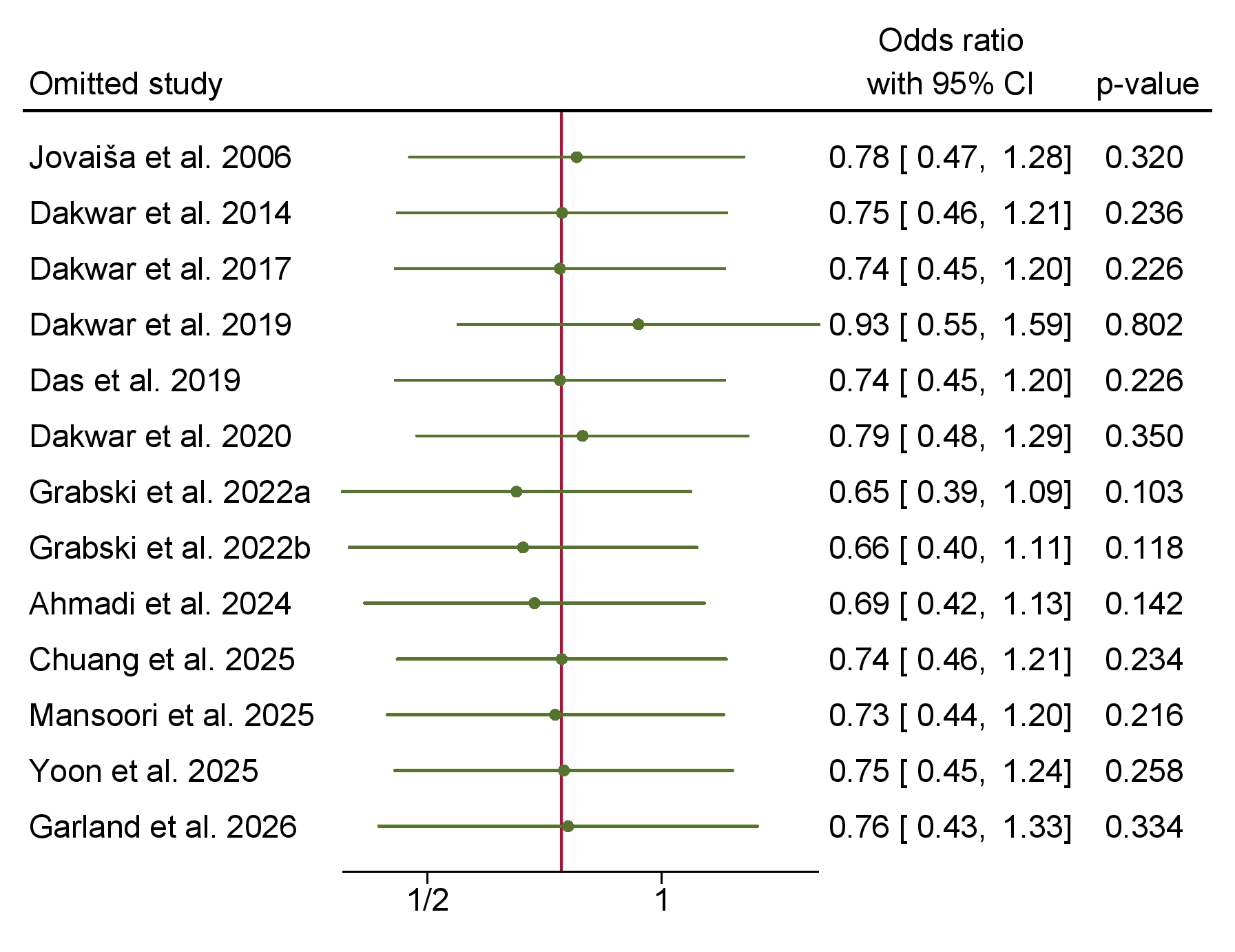


**Supplementary Figure** **S13**. Sensitivity analysis of serious adverse events.


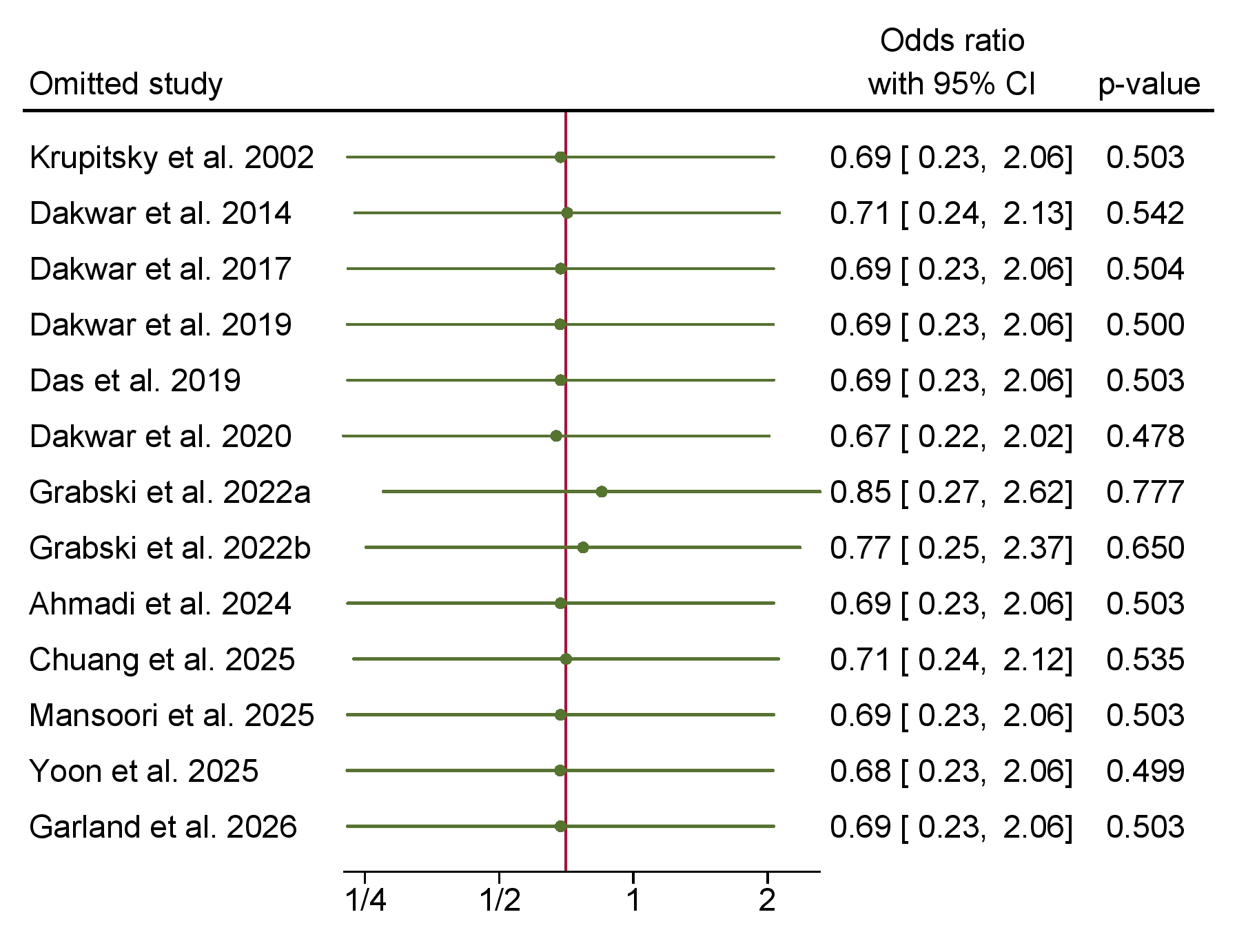


**Supplementary Figure** **S14**. Sensitivity analysis of other adverse events.


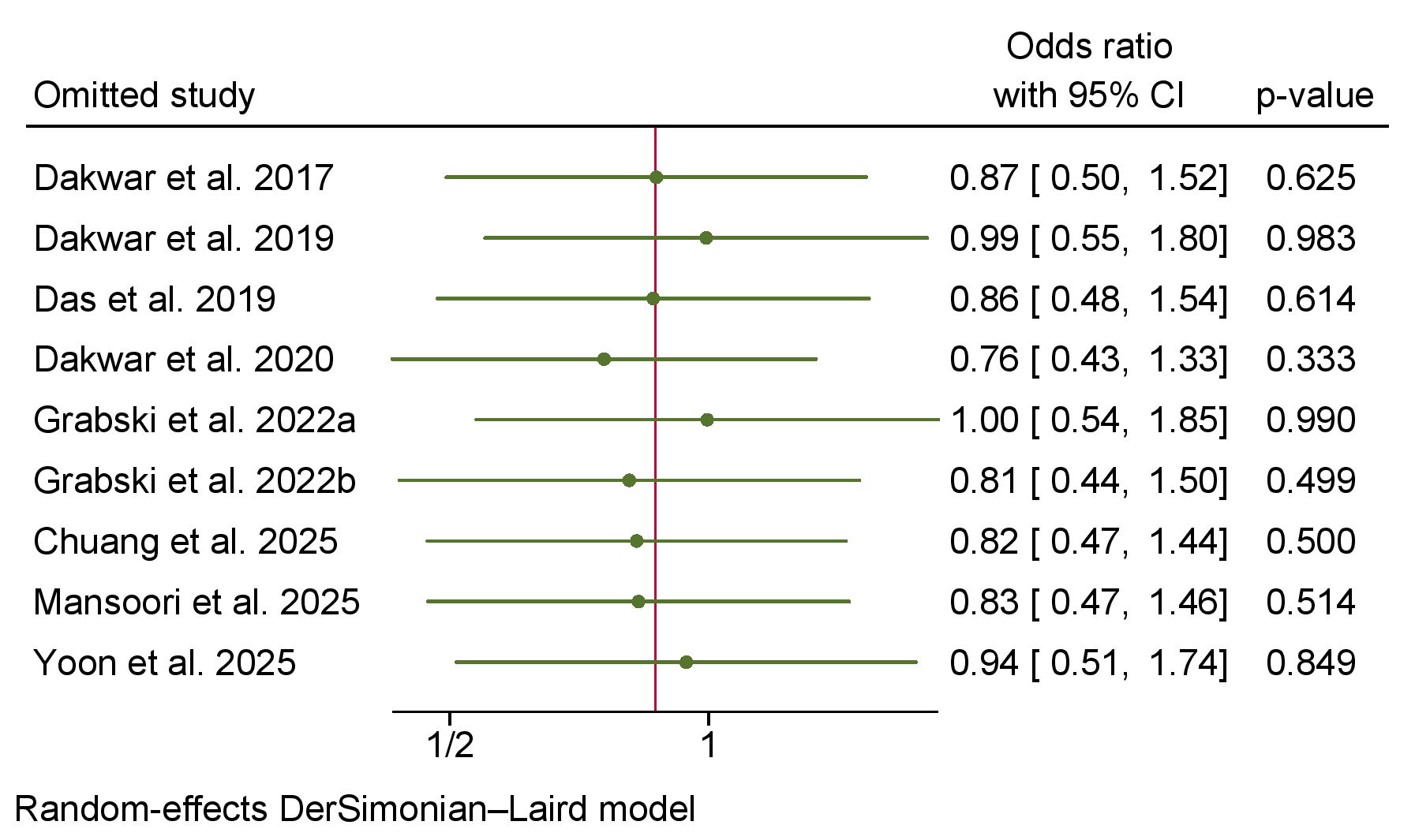


**Supplementary Figure** **S15.** Funnel plot for assessing the publication bias for abstinence rates.


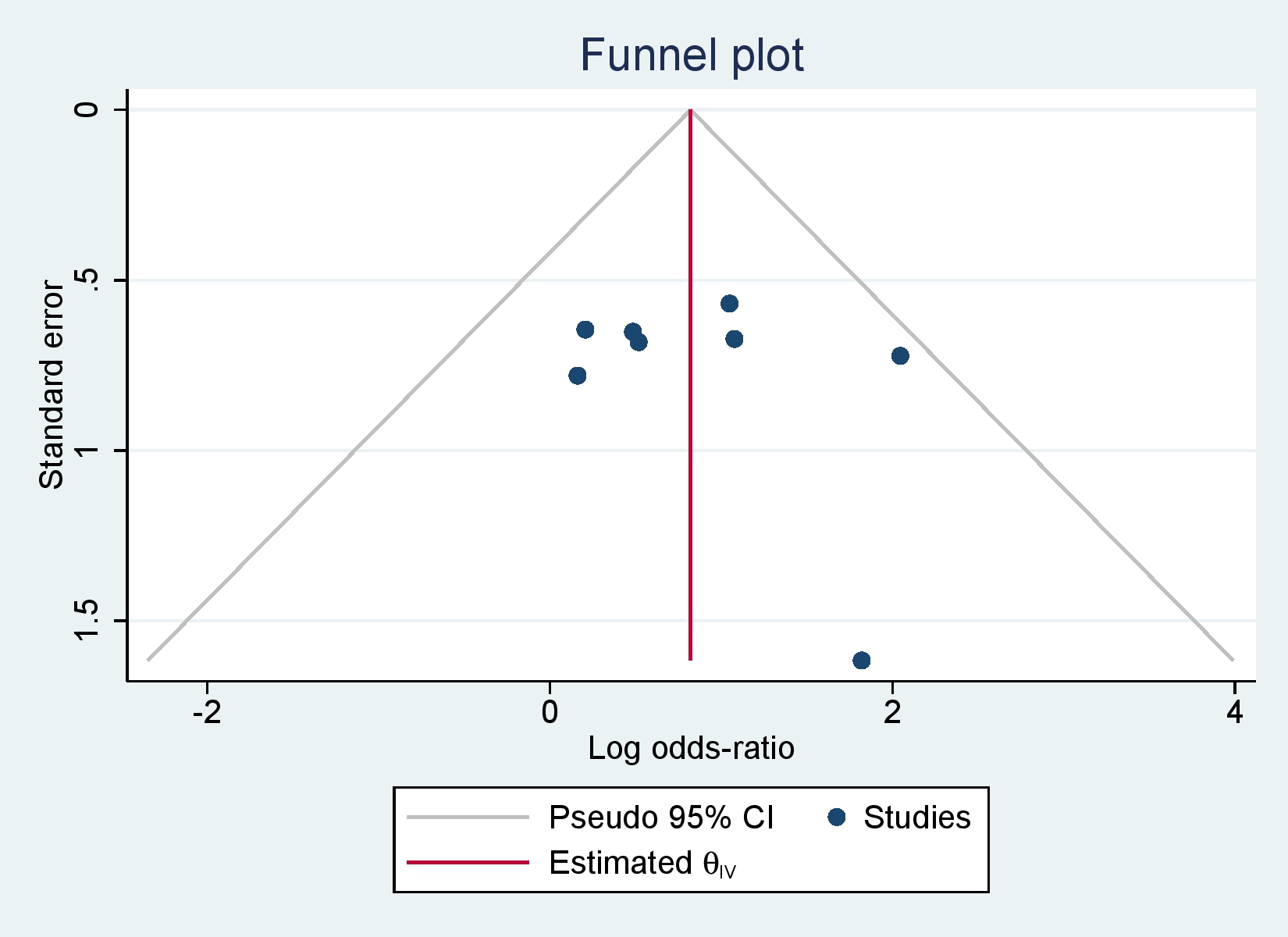


**Supplementary Figure** **S16.** Funnel plot for assessing the publication bias for dropout rates.


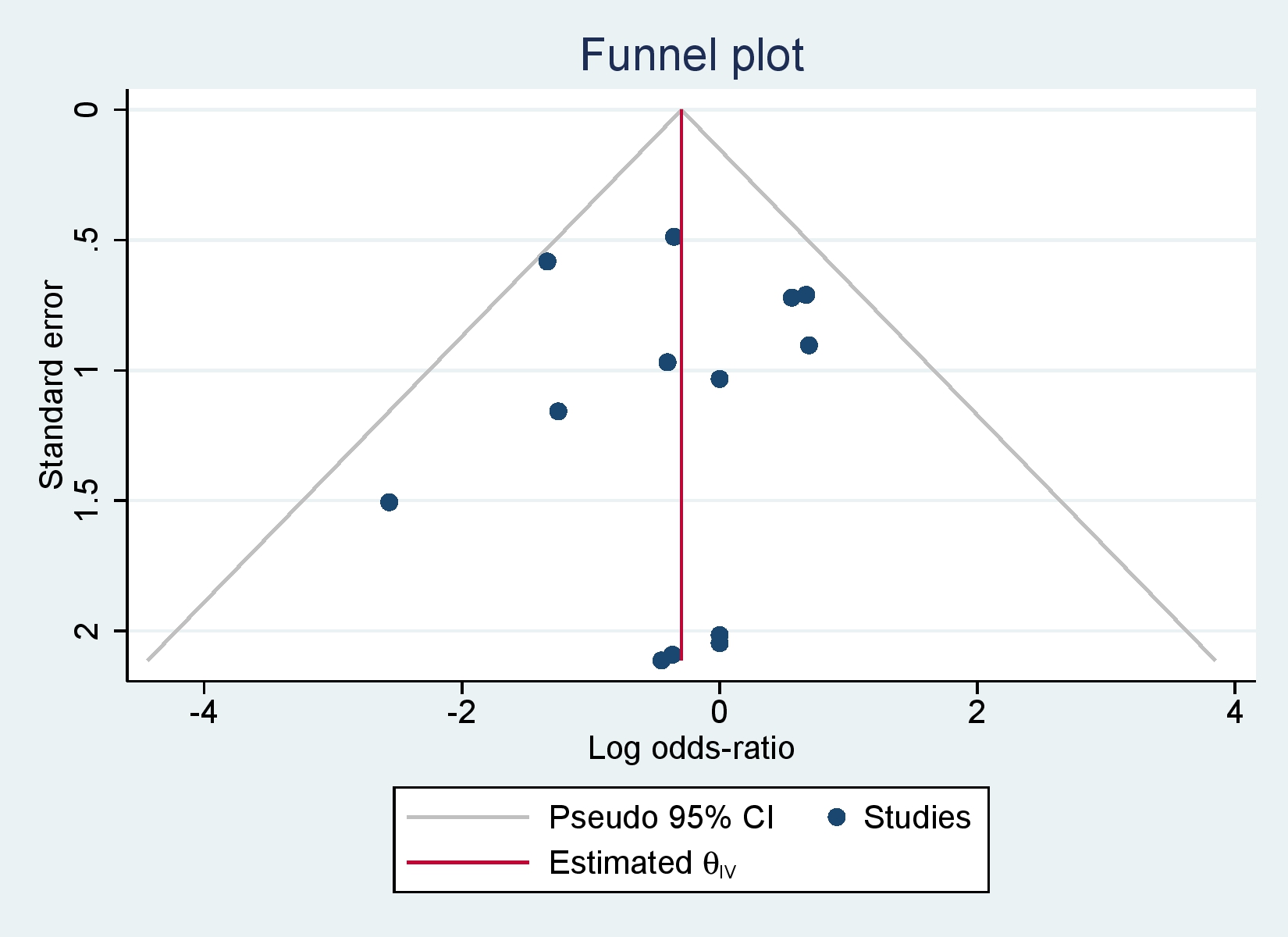


**Supplementary Figure** **S17.** Funnel plot for assessing the publication bias for serious adverse events.


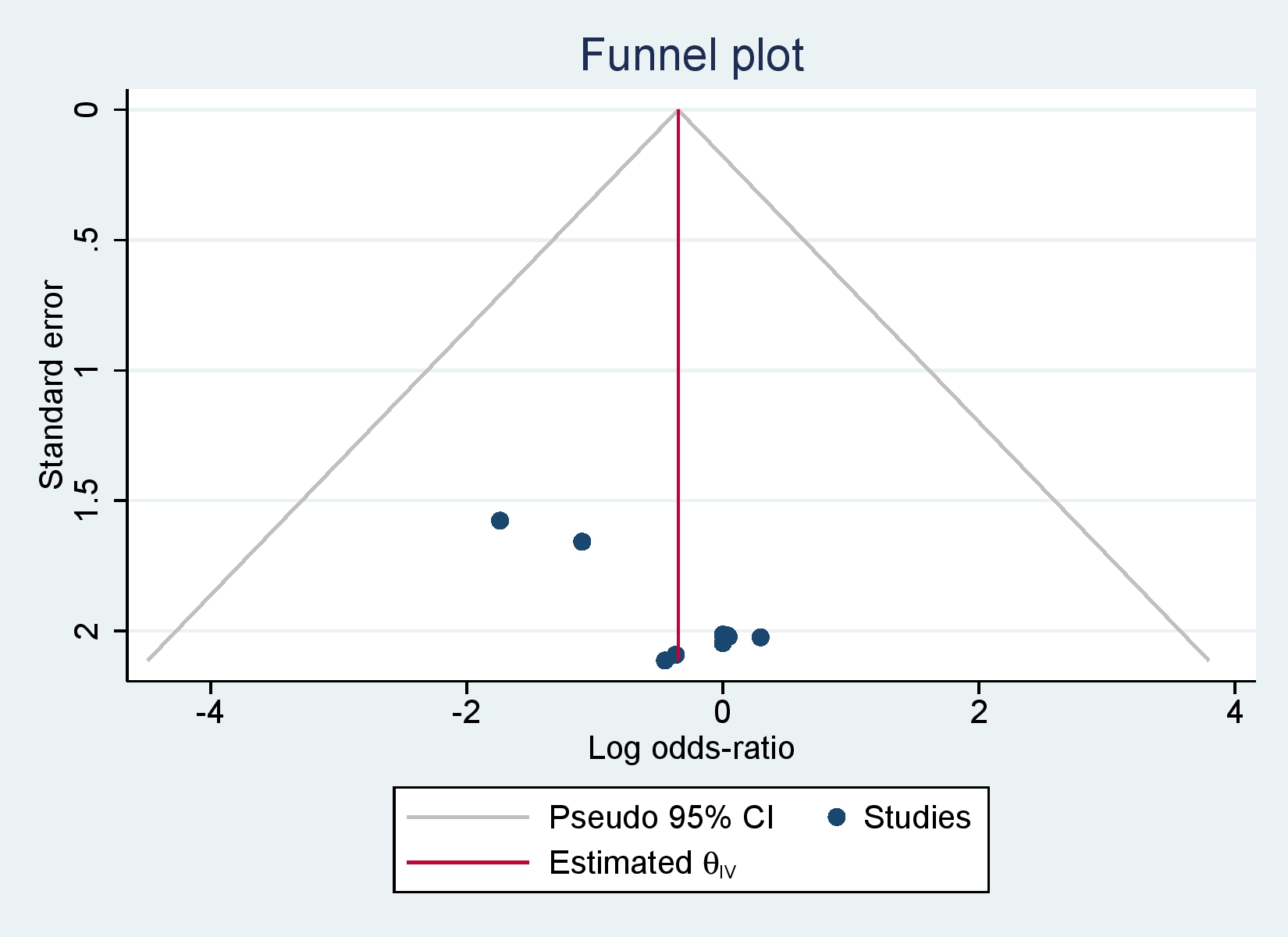


**Supplementary Figure** **S18.** Funnel plot for assessing the publication bias for other adverse events.


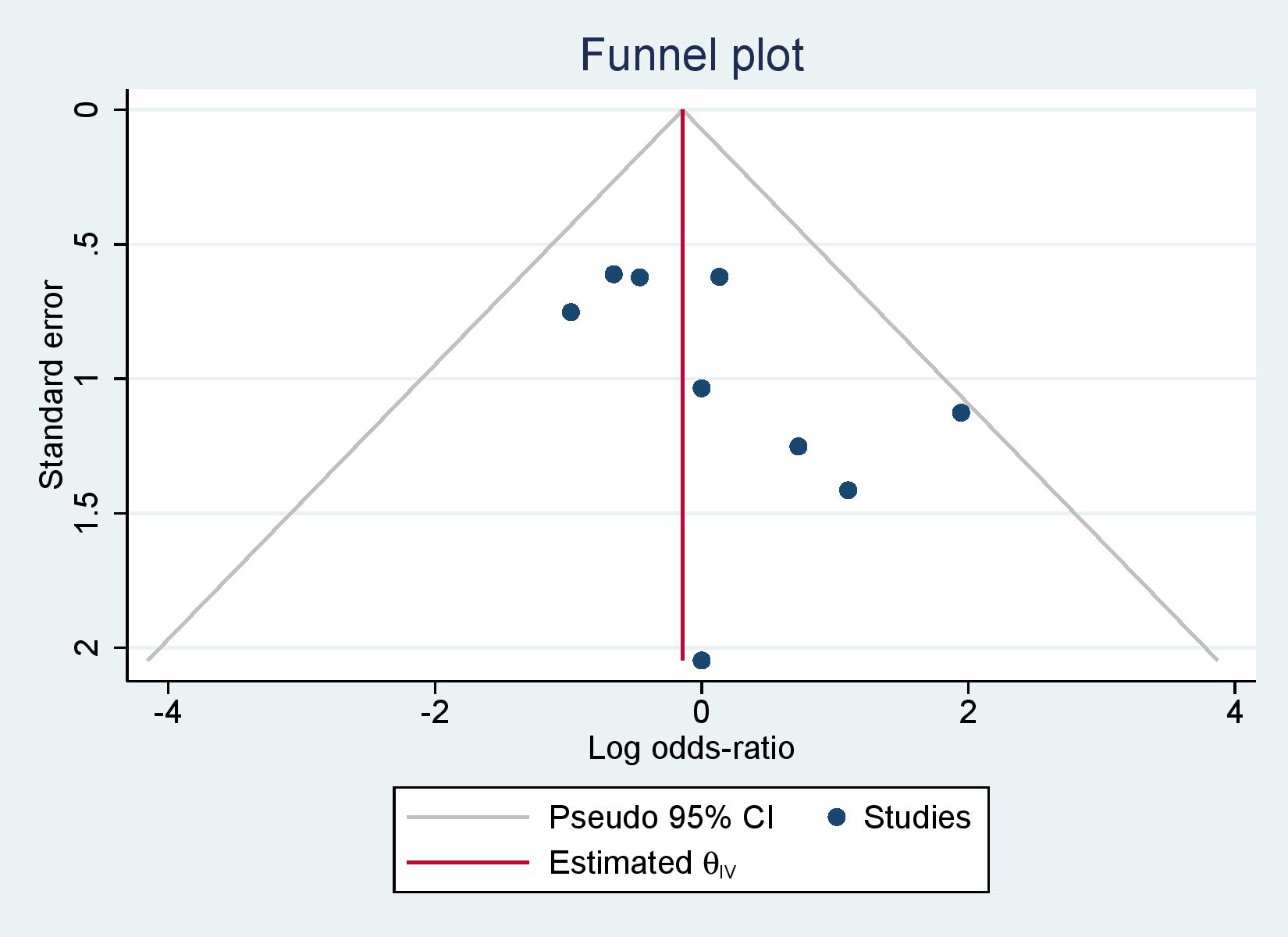

Supplement: Supplementary file 1 [file SupplementaryFile1.docx]
